# Synthesis and Property Detection of the Ho_2_BiNbO_7_/ZnBiTmO_4_ Composite Catalyst for Photocatalytic Degradation of Brilliant Green

**DOI:** 10.3390/nano16150951

**Published:** 2026-08-02

**Authors:** Jingfei Luan, Boyang Liu

**Affiliations:** 1 School of Physics, Changchun Normal University, Changchun 130032, China; boyangliu152@outlook.com; 2 State Key Laboratory of Pollution Control and Resource Reuse, School of the Environment, Nanjing University, Nanjing 210093, China

**Keywords:** Z-scheme Ho_2_BiNbO_7_/ZnBiTmO_4_ heterojunction photocatalyst, degradation mechanism, brilliant green, visible light irradiation, catalytic degradation velocity, degradation pathway

## Abstract

A high-performance Z-scheme Ho_2_BiNbO_7_/ZnBiTmO_4_ heterojunction (HZ) photocatalyst was prepared for the first time using a wet impregnation method. The HZ photocatalyst significantly improved the separation efficiency of the photoinduced electrons and the photoinduced holes; meanwhile, the HZ photocatalyst could effectively broaden the visible light spectrum via a specific mechanism of the Z-scheme heterojunction structure. The experimental results displayed that the HZ photocatalyst had strong catalytic activity when the brilliant green (BLG) was degraded. In particular, the degradation rate of BLG when using the HZ photocatalyst was found to be 99.47%, and the mineralization efficiency of the total organic carbon (TOC) concentration was found to be 98.26% when using the HZ photocatalyst under visible light irradiation (VILIIR). The HZ photocatalyst possessed higher photocatalytic activity compared with Ho_2_BiNbO_7_, ZnBiTmO_4_, or N-doped TiO_2_ (N-T). The degradation rate of BLG when using the HZ photocatalyst was 1.27 times higher than that when using Ho_2_BiNbO_7_, 1.15 times higher than that when employing ZnBiTmO_4_, or 2.91 times higher than that when using N-T under VILIIR. The mineralization efficiency of the TOC concentration after catalytic degradation of BLG when employing the HZ photocatalyst was 1.31 times higher than that when employing Ho_2_BiNbO_7_, 1.19 times higher than that when employing ZnBiTmO_4_, or 3.14 times higher than that when using N-T under VILIIR. The experimental generating radicals confirmed that the HZ photocatalyst might produce diverse reactive radicals, which contained superoxide anions (•O_2_^−^), hydroxyl radicals (•OH) and photogenerated holes (h^+^) after catalytic degradation of BLG. The descending order of oxidizing capacity for above three radicals was as follows: •OH > •O_2_^−^ > h^+^. The descending order of the photocatalytic activity for the four photocatalysts was as follows: HZ > ZnBiTmO_4_ > Ho_2_BiNbO_7_ > N-T. The intermediate degradation products of BLG were detected by employing the HZ photocatalyst during the photocatalytic degradation process of BLG; the reliability, reusability, and stability of the HZ photocatalyst were proven by quintic cyclical degradation experiments of BLG. This study developed the degradation pathways and degradation mechanism of BLG when using the HZ photocatalyst under VILIIR. This work supplies novel thought for the design and manufacture of Z-scheme heterojunction catalysts, and it provides a basis for developing an efficient environmental remediation technique for BLG pollution.

## 1. Introduction

The world is currently facing severe challenges of drinking and domestic water scarcity. Water pollution stands as one of the most critical ecological threats today. Brilliant green (BLG) is primarily utilized in biological staining, bacterial culture differentiation, and as a pH indicator. Therefore, BLG is widely utilized in developing regions or traditional industrial zones. However, BLG exhibits moderate acute toxicity, along with clear mutagenic effects and potential carcinogenic and teratogenic risks. Humans can be exposed to BLG either through skin contact (where the dye has been shown to penetrate within 30 s–300 s from dyed paper products) or through the food chain via dye transfer from packaging or contaminated materials. Environmentally, the BLG is not readily biodegradable and exhibits high aquatic toxicity. The complex molecular structure of the BLG makes the BLG resistant to conventional wastewater treatment, resulting in severe water pollution with toxic, mutagenic, and carcinogenic effects on both aquatic ecosystems and human health [1,2]. Consequently, the development of efficient technologies and methods for removing BLG from industrial wastewater possesses significant practical importance and environmental urgency.

Conventional methods, such as coagulation, flocculation, adsorption, and membrane technology, exhibit extremely low efficiency for the complete conversion of BLG, and ultimately, other secondary wastes are generated. Consequently, the above traditional water treatment approaches are incapable of safely degrading BLG in industrial wastewater.

Photocatalytic advanced oxidation technology, which is regarded as an emerging mainstream method for water environment purification, has attracted growing attention from researchers. The photocatalytic advanced oxidation process has potential owing to its low cost, high efficiency, lower energy cost, environmental friendliness, and ability to completely decompose organic pollutants in wastewater [3]. However, conventional photocatalysts such as metal oxide ZnO and TiO_2_ face challenges in practical applications, which include a wider bandgap, high recombination efficiency of photogenerated electrons and photogenerated holes, and less active sites [4,5]. In order to address these challenges, the evolution of new photocatalysts, which have expeditious visible light responsiveness, a high carrier separation efficiency, and a high specific area, has become a central research focus in the field of photocatalytic oxidation processes [6].

A_2_B_2_O_7_-type oxides, which have a pyrochlore structure, and AB_2_O_4_-type oxides, which have a spinel structure, exhibit significant potential for the effective degradation of various organic pollutants [7]. This is owing to their excellent thermal stability, chemical stability, strong visible light response, and abundant active sites. Dongdong Lv et al. successfully prepared Bi_2_Sn_2_O_7_ via a hydrothermal method and investigated the catalytic property of Bi_2_Sn_2_O_7_ for degrading rhodamine B [8]. Junyu Lang et al. prepared Sn_2_Ta_2_O_7_ by employing an ordinary hydrothermal synthesis method and employed Sn_2_Ta_2_O_7_ for degrading methyl orange under visible light irradiation (VILIIR) [9]. Derkaoui Khaled et al. prepared the photocatalyst MnFe_2_O_4_ using the coprecipitation method and studied the catalytic activity of MnFe_2_O_4_ for the degradation of rhodamine B under VILIIR [10]. Muthukrishnaraj A et al. prepared CuBi_2_O_4_ via a novel single-step solvothermal process and evaluated the effectiveness of CuBi_2_O_4_ for degrading methylene blue under VILIIR [11].

Previous studies had demonstrated that the photocatalytic activity of photocatalysts could be enhanced by doping with different elements. For example, Phuruangrat et al. prepared Ho-doped zinc oxide, and Ho-doped ZnO was observed to possess a significant improvement in the catalytic degradation efficiency of the subunit methylene blue compared with pure ZnO [12]. Zhu Xi Miao et al. prepared Bi-doped TiO_2_ via a sol–gel method, and it exhibited a markedly higher photocatalytic degradation efficiency for methyl orange than pure TiO_2_ [13]. Yang Jikai et al. successfully prepared Nb-doped TiO_2_ using an ultrasonic spray pyrolysis method, and it showed a superior photocatalytic activity for the degradation of methylene blue compared with TiO_2_ under VILIIR [14]. Additionally, Varadharajan Krishnakumar et al. prepared Zn-doped TiO_2_ by employing a sol–gel method, and the degradation rate of reactive yellow 145 by employing Zn-doped TiO_2_ was considerably higher than that by employing pure TiO_2_ under VILIIR [15]. Karnchana N et al. prepared a Tm-doped ZnO nanocatalyst via a tartaric acid-assisted combustion method, and it exhibited higher photocatalytic activity for the degradation of methylene blue compared with the pure ZnO nanocatalyst under VILIIR [16]. Inspired by the above studies, in this work, we designed and synthesized two novel visible light-responsive photocatalysts, Ho_2_BiNbO_7_ and ZnBiTmO_4_; subsequently, the holmium element, bismuth element, niobium element, zinc element, and thulium element were incorporated into the oxide frameworks.

Although the single-component pyrochlore oxide or the single-component spinel oxide exhibited good stability, the rapid recombination of the photogenerated electron–hole pairs and insufficient active sites within the bulk phase of the above oxides could severely limit their overall photocatalytic degradation efficiency and mineralization capability for removing organic pollutants. In order to fill the above-mentioned scientific gap, this study provided the direct Z-scheme charge transfer mechanism for addressing this challenge. Constructing heterojunction photocatalysts could generate an intrinsic electric field between a couple of component single-phase catalysts of a binary heterojunction [17]. The mechanism effectively facilitated the separation efficiency of the photogenerated electron–hole pairs; meanwhile, a high redox potential was maintained. Therefore, more efficient generation of active radicals, which included hydroxyl radicals (•OH), superoxide anions (•O_2_^−^) and photoinduced holes (h^+^), was realized. Moreover, the above active radicals possessed strong oxidizing capability.

The Ag/TiO_2_ catalyst prepared by Fattah W.I.A. et al. obtained a removal rate of 75% when degrading chlorpyrifos after VILIIR for 120 min; the degradation rate of chlorpyrifos when using the Ag/TiO_2_ catalyst was considerably higher than that when using the individual Ag component or the individual TiO_2_ component [18]. Wan-Kuen Jo et al. prepared a direct Z-type g-C_3_N_4_/TiO_2_ when using a wet impregnation method and employed g-C_3_N_4_/TiO_2_ for the photocatalytic degradation of isoniazid (an anti-tuberculosis drug) under VILIIR, as a result, the experimental results revealed that after VILIIR for 240 min, the photocatalytic removal rate of isoniazid reached 90.8%, 73.3% or 13.5% when using g-C_3_N_4_/TiO_2_, outstanding TiO_2_ nanotubes or g-C_3_N_4_ [19]. According to these results, the direct Z-type Ho_2_BiNbO_7_/ZnBiTmO_4_ heterojunction (HZ) photocatalyst powder was designed and prepared in this study. This specific Z-scheme heterojunction combination for Ho_2_BiNbO_7_ and ZnBiTmO_4_ had not been reported previously.

In this study, the adjustment of the band structure of HZ obtained efficient separation of photogenerated electron–hole pairs, and concurrently, the light absorption scope of HZ was broadened to the visible light range. In the experiment, HZ exhibited an excellent catalytic degradation property toward typical poisonous organic pollutants, such as BLG contained in industrial wastewater. In recent years, ZnS quantum dots (with a removal efficiency of 88% under visible light irradiation) have been explored for the photocatalytic degradation of BLG [1]. This research not only demonstrates higher photocatalytic activity and innovative structural design of the HZ photocatalyst for efficient degradation of organic pollutants but also provides experimental evidence and an academic foundation for facilitating the practical application of visible-light-driven photocatalysis technology in water environmental remediation.

## 2. Experiment and Methods

### 2.1. Materials and Chemical Agents

Aladdin Group Chemical Reagent Co., Ltd. (Shanghai, China) provided HoCl_3_ (purity quotient = 99.95%), NbCl_5_ (purity quotient = 99.995%), Bi(NO_3_)_3_·5H_2_O (purity quotient = 99.995%), Zn(NO_3_)_2_·6H_2_O (purity quotient = 99.998%), Tm(NO_3_)_3_·5H_2_O (purity quotient = 99.9%), isopropylcarbinol (IPA, C_3_H_8_O, purity quotient ≥ 99.999%) and BLG (C_27_H_34_N_2_O_4_S, purity quotient ≥ 98%). Sodium citrate dihydrate (C_6_H_5_Na_3_O_7_·2H_2_O, purity quotient = 99%) was bought from Sinopharm Group Chemical Reagent Co., Ltd. (Shanghai, China). Ethylenediaminetetraacetic acid (EDTA, C_10_H_16_N_2_O_8_, purity quotient = 99.995%) was bought from Merck Group Chemical Reagent Co., Ltd. (Shanghai, China). Benzoquinone (BQ, C_6_H_4_O_2_, purity quotient ≥ 99.5%) was bought from Macklin Biochemical Co., Ltd. (Shanghai, China). The reagent chemical was utilized as experimental requirements without modification.

### 2.2. Preparation of Ho_2_BiNbO_7_

The Ho_2_BiNbO_7_ photocatalyst was prepared via a rapid microwave-assisted high-temperature treatment method. A mixture of 10 × 10^−3^ mol HoCl_3_, 5 × 10^−3^ mol NbCl_5_ and 5 × 10^−3^ mol Bi(NO_3_)_3_·5H_2_O was dissolved in 100 mL of distilled water and magnetically stirred at room temperature for 30 min. The pH value of the resultant mixture was adjusted to 12 by adding a sodium hydroxide solution [20]. The pH value of 12 was based on previous studies on the synthesis of the relevant samples, which should exist in an alkaline environment [20]. The solution was then transferred into a 150 mL vessel and heated in a microwave oven at a power of 750 W for 45 min. After microwave treatment, the mixed solution was allowed to cool naturally to room temperature. The obtained precipitate was isolated via centrifugation and thoroughly washed with distilled water; then, the precipitate was liquified with ethanol and desiccated at 70 °C for 24 h. Finally, the prepared photocatalyst underwent two annealing treatments, through which the Ho_2_BiNbO_7_ photocatalyst was obtained [20].

### 2.3. Preparation of ZnBiTmO_4_

The ZnBiTmO_4_ photocatalyst was prepared via a homogeneous precipitation-assisted heat treatment method. Zn(NO_3_)_2_·6H_2_O, Bi(NO_3_)_3_·5H_2_O, and Tm(NO_3_)_3_·5H_2_O were dissolved in 800 mL of deionized water or mineral acid, and the resultant solution was magnetically stirred for 180 min. Subsequently, 1 mmol of surfactant sodium citrate dihydrate (C_6_H_5_Na_3_O_7_·2H_2_O) was added to the mixed solution. The urea was slowly added while the mixed solution was stirred continuously, and a homogeneous mixture was formed with the complete addition of the urea. The homogeneous mixture was heated to 95 °C with constant stir operation and maintained at 95 °C for 2 h. The obtained precipitate was stored in a desiccator. The dried solid was then annealed in a muffle furnace at 450 °C for 2 h under an air atmosphere, where the heating rate was 10 °C/min. Ultimately, the ZnBiTmO_4_ photocatalyst was obtained [21].

### 2.4. Production Process of HZ

**Figure 1:**
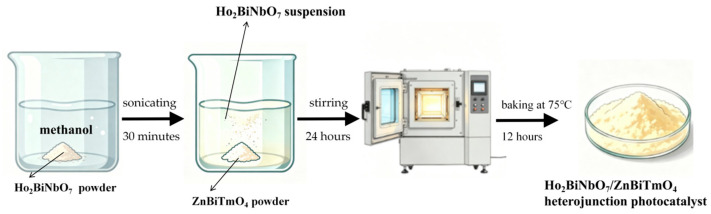
Schematic preparation route for Ho_2_BiNbO_7_/ZnBiTmO_4_ heterojunction photocatalyst.

In this work, the HZ catalyst was prepared by employing a wet dipping method. The Ho_2_BiNbO_7_ (0.008 mol) powder was dispersed in methanol (50 mL) and sonicated for 30 min. Subsequently, the ZnBiTmO_4_ (0.008 mol) catalyst was added to the Ho_2_BiNbO_7_ suspension, which was stirred at room temperature for 24 h. Finally, the obtained product was baked at 75 °C for 12 h. Ultimately, the HZ photocatalyst was obtained [22]. Figure 1 illustrates the preparation process of the Ho_2_BiNbO_7_/ZnBiTmO_4_ heterojunction photocatalyst.

### 2.5. Method for Preparing N-Doped TiO_2_

The titanium dioxide powder was deposited on a platina sheet and put into a tube furnace. Initially, a mixture of NH_3_/Ar gas with a volume ratio of 3:2 was adopted at a flowing speed of 50 mL/min for two hours, during which air and moisture were purged. When the airflow underwent stabilization with a flow rate of 200 mL/min, the temperature was raised from room temperature to 600 °C at a heating rate of 10 °C/min; then, the temperature was maintained at 600 °C for 6 h under the continuous flow of the NH_3_/Ar gas mixture. The system was then cooled to room temperature at a cooling rate of 20 °C/min, while the same gas flow rate was maintained. The final product was repossessed and underwent the grinding treatment for obtaining the N-doped TiO_2_ photocatalyst [23].

### 2.6. Photoelectrochemical Experiment

The photoelectrochemical properties of Ho_2_BiNbO_7_, ZnBiTmO_4_ and HZ were detected using a CHI-660D galvanochemistry active station (Chenhua Instruments Co., Ltd., Shanghai, China). The working station was composed of three electrodes: we used the platinum electrode as the counter electrode, silver/silver chloride (AgCl) as the reference electrode, and the prepared conductive substrate coated with a catalyst as the operating electrode. The sodium sulfate solution was utilized as an electrolyte with a concentration of 0.3 mol/L. We scattered 2.5 mg of photocatalyst powder into 250 microliters of a mixed solvent of ethanol and ethylene glycol with a volume ratio of 1:1. Subsequently, we performed ultrasonic processing for 40 min and magnetic stirring for three hours to gain a uniform suspension. In rapid sequence, 30 microliters of the suspended liquid were dropwise coated on the surface of a pretreating glass carbon electrode, which had a glass carbon electrode area of 0.07 cm^2^ (around 0.3 mg/cm^2^). The operating electrode was prepared by drying at room temperature for 30 min. In order to study the electrochemical performance of the photocatalysts systematically, electrochemistry resistance spectrogram examination was carried out in potassium chloride solution. All examination was performed at room temperature.

### 2.7. Experimental Setup and Procedure

The photocatalytic activity of three different photocatalysts (Ho_2_BiNbO_7_, ZnBiTmO_4_ and HZ) for degrading the BLG was estimated by employing a CEL-LB70 photochemistry reactive instrument (CEL-LB70, Zhongjiao Jinyuan Technology Co., Ltd., Beijing, China). Firstly, 0.2 g of photocatalyst grinding powder of Ho_2_BiNbO_7_, ZnBiTmO_4_ or HZ was added to 300 mL of the BLG solution at a concentration of 0.03 mmol/L. Prior to the occurrence of the catalytic reaction, the mixed solution was stirred in the dark for 45 min to reach adsorption–desorption equilibrium. In order to determine the appropriate dark stirring time for establishing adsorption–desorption equilibrium of the BLG by utilizing the HZ, a preliminary kinetic experiment was accomplished under the identical experimental conditions prior to the formal photocatalytic degradation measurements of the BLG. Table S1 shows the concentration variation in the brilliant green by the adsorption effect of the HZ under dark conditions. It can be seen in Table S1 that the BLG concentration was monitored at regular intervals (every 9 min) during the dark stirring process for up to 63 min without visible light irradiation. The results showed that the BLG concentration plateaued at approximately 45 min; as a result, the BLG concentration maintained a slight change from 46 min to 63 min during the dark stirring process. The above result confirmed that the adsorption–desorption equilibrium was effectively established within this dark adsorptional timeframe of 45 min. Accordingly, the above process ensured that the photocatalyst particles of Ho_2_BiNbO_7_, ZnBiTmO_4_ or HZ were uniformly distributed within the reactive solution; meanwhile, the above process helped the BLG molecule to reach the adsorption–desorption equilibrium status on the photocatalyst surface. Clearly, this process may differentiate and rule out the effect of physical adsorption on the degradation efficiency of the BLG. Therefore, the subsequent catalytic degradation data indicated the photocatalytic activity of the catalysts.

The catalytic degradation reaction was completed under VILIIR using a 500 W xenon lamp, which was composed of a 420-nanometer filter as the light source. During the photocatalytic reactive process, 5 mL of the reactive solution was gathered every 20 min. After centrifugation at 7200 rpm for 10 min, the supernatant was extracted for further analysis by employing an Agilent 200 high-performance liquid chromatograph system (Agilent Technologies, Palo Alto, CA, USA).

In order to study the mineralizing conduct of the TOC systematically in the process of degradation for the BLG, a TOC analysis meter (TOC-5000 A, Shimadzu Corporation, Kyoto, Japan) was utilized to analyze the mineralizing efficiency of the TOC-C during the degradation process of the BLG. In the process of the experimental testing, potassium biphthalate (KHC_8_H_4_O_4_) standard water solution was utilized as the adjusting standard reagent. A standard curve was plotted against carbonic concentration, which changed from 0 mg/L to 100 mg/L.

In order to further investigate the degradation intermediate products, a Thermo Quest LCQ Duo liquid chromatograph and mass spectrograph (LC-MS) (Thermo Fisher Scientific Corporation, Waltham, MA, USA) was utilized for identification analysis of the intermediate products during the degradation process of the BLG. After the catalytic reaction, 20 microliters of the specimen were extracted from the reactive solution and syringed into the LC-MS instrument, which contained a Beta Basic-C18 chromatographic column. Methanol and deionized water with a volume ratio of 60:40 were used as the mobile phase, and the floating rate of the mobile phase was maintained at 0.2 mL/min. The mass spectrometer was utilized to test the mass-to-charge ratio (*m*/*z*), which changed from 50 to 500. Then, the daughter ion snipping was investigated to clarify the possible degradation routes and intermediate products of the BLG.

### 2.8. Characterization Instrument

Crystal data were obtained using a Shimadzu XRD-6000 diffraction instrument (Kyoto, Japan). Fourier transform infrared spectroscopy (FTIR) spectrograms were obtained using a WQF-530A spectrograph (Beifen-Ruili Analytical Instrument (Group) Co., Ltd., Beijing, China) to clarify the molecular structure, chemical bond styles and functional group constituents of the photocatalysts. The Raman spectrograms were generated by employing INVIA0919-06 equipment supplied by RENSHAW plx (Wotton-under-Edge, Gloucestershire, UK) to analyze the chemical structure, crystal kinetics, and molecular interaction of the photocatalysts. The surface appearance and crystal lattice stripes of the photocatalysts were analyzed using a TEM (Talos F200X G2 equipment, Thermo Fisher Scientific, Waltham, MA, USA), while EDS spectrograms were performed to detect the elemental distribution and contents of the photocatalysts. An XPS (PHI 5000 VersaProbe instrument, ULVAC-PHI, Maoqi City, Japan) was used to analyze the surface elemental valence and surface elemental content of the photocatalysts. The XPS data analysis was performed using XPSPEAK software. All XPS spectra were calibrated for charging effects by setting the binding energy of the carbon C 1s peak, while a Shirley-type background was applied when fitting the XPS data from Ho_2_BiNbO_7_, ZnBiTmO_4_ and HZ. The optical performance of the specimens was estimated by employing an ultraviolet and visible spectrophotometer. Moreover, the PL spectrograms and fluorometric lifetimes of the photocatalysts were examined using an FLS980 fluorescence spectrophotometer (Edinburgh Instruments Ltd., Edinburgh, UK). The excitation wavelength of the specimen was 300 nanometers in the PL experiment. Ultimately, the activity radicals from the specimens were tested by employing an EPR spectrometer with A300 equipment (Bruker Corporation, Karlsruhe, Germany).

Additionally, Escalab 250 xi equipment (Thermo Fisher Scientific, Waltham, MA, USA) was utilized to measure the ionizing potential of the valence band for the photocatalyst by employing a UPS. Figure S1 shows the research process for each stage of this study.

## 3. Results and Discussion

### 3.1. Property Characterization of Photocatalysts

The phase structure and phase compositions of the prepared Ho_2_BiNbO_7_ photocatalyst, ZnBiTmO_4_ photocatalyst, and the Z-scheme HZ photocatalyst were analyzed with X-ray diffraction (XRD) measurement. Figure S2a displays the XRD plot and Pawley simulated result of Ho_2_BiNbO_7_. Figure S2b exhibits the atomic structure of Ho_2_BiNbO_7_. According to Figure S2a, the experimental result indicated that the prepared Ho_2_BiNbO_7_ had distinct sharp diffraction peaks. The Pawley method of Materials Studio software version-2023 was utilized to refine the experimental XRD data of Ho_2_BiNbO_7_, and many parameters underwent optimization. The simulated result in Figure S2a shows that the Rp factor of Ho_2_BiNbO_7_ was 4.22%; meanwhile, Ho_2_BiNbO_7_ remained in the isometric system with the pyrochlore structure. Moreover, the space group of Ho_2_BiNbO_7_ was Fd3m, and subsequently, the crystal cell parameters of Ho_2_BiNbO_7_ were a = b = c = 10.622091 Å; the bulk of the unit cell Ho_2_BiNbO_7_ was 1198.48 (Å)^3^. The interplanar distance from the crystal plane indices of (222) for Ho_2_BiNbO_7_ was 0.307 nm. The corresponding atomic coordinates and the structural parameters of Ho_2_BiNbO_7_ are shown in Table S2. The simulation result of Ho_2_BiNbO_7_ illustrated that the observational experimental intensity was consistent with the simulated intensity in the cubic crystal system pyrochlore structure. Sharp diffractive peaks could be seen in the Ho_2_BiNbO_7_ plot, and concurrently, the diffractive peaks of the impure phases could not be detected. These results proved that the prepared Ho_2_BiNbO_7_ photocatalyst possessed a high degree of crystallinity; meanwhile, impure phases were not present in Ho_2_BiNbO_7_. These results also laid a good foundation for the successful preparation of a pure HZ photocatalyst with high photocatalytic activity. The structure of Ho_2_BiNbO_7_ is revealed in Figure S2b. According to the structure of Ho_2_BiNbO_7_, the Bi–O bond length was 2.29497 Å. The crystal structure of Ho_2_BiNbO_7_ highlighted two diverse types of Ho–O bonds: the long Ho–O bond, which had a bond length of 2.642 Å, and the cutty Ho–O bond, which possessed a bond length of 2.300 Å. The distortion observed within the MO_6_ octahedra (M = Bi^3+^ and Nb^5+^) of Ho_2_BiNbO_7_ indicated a distortion in the crystal structure of Ho_2_BiNbO_7_; as a result, the recombination rate of the photogenerated electron–hole pairs could be depressed. Therefore, the photocatalytic activity of Ho_2_BiNbO_7_ could be increased, according to above mentioned studies. In addition, aforementioned research studies clarified that the catalytic activity of Ho_2_BiNbO_7_ was improved while the M-O-M bond angle approached 180 degrees. According to the centrical oxygen atom, the Bi-O-Bi bond angle from Ho_2_BiNbO_7_ was 110.778°, and accordingly, Ho_2_BiNbO_7_ possessed a better photocatalytic activity [24,25].

Figure S3a represents the XRD plot and the Pawley simulated result of ZnBiTmO_4_. Figure S3b exhibits the atomic structure of ZnBiTmO_4_. According to Figure S3a, the prepared ZnBiTmO_4_ had evident sharp diffractive peaks. The Pawley method of Materials Studio software was utilized to refine the experimental XRD data of ZnBiTmO_4_, and many parameters underwent optimization. According to the simulated result in Figure S3a, the Rp factor of ZnBiTmO_4_ was 6.54%, and ZnBiTmO_4_ remained in the body-centered tetragonal system with a spinel structure. Moreover, the space group of ZnBiTmO_4_ was I41/AMD; subsequently, the crystal cell parameters in ZnBiTmO_4_ were a = b = 14.798828 Å, c = 9.632210 Å, and ultimately, the bulk of the unit cell ZnBiTmO_4_ was 2109.51 (Å)^3^. The interplanar distance from the crystal plane indices of (103) for ZnBiTmO_4_ was 0.314 nm. The corresponding atomic coordinates and the structural parameters of ZnBiTmO_4_ are shown in Table S3. The ultimate refinement results of ZnBiTmO_4_ illustrated that the observed experimental intensity was in good agreement with the calculated intensity in the tetragonal system spinel structure. Sharp diffraction peaks were present in the pattern of ZnBiTmO_4_, and concurrently, the diffraction peaks of the impure phases were not detected. These results proved that the prepared ZnBiTmO_4_ photocatalyst possessed a high degree of crystallinity; meanwhile, impure phases were not found in ZnBiTmO_4_. The results also laid a good foundation for the preparation of a pure HZ photocatalyst with high photocatalytic activity. The structure of ZnBiTmO_4_ is revealed in Figure S3b. According to the structure of ZnBiTmO_4_, the bond length of the Zn–O bond was 2.27640 Å; meanwhile, the Bi–O(2) bond had a bond length of 2.59429 Å, and the Bi–O(3) bond had a bond length of 2.21634 Å. The structure of ZnBiTmO_4_ was distorted due to two different lengths of the Bi–O bond; thus, the recombination efficiency of the photogenerated electrons and photogenerated holes could be depressed. Therefore, the photocatalytic activity of ZnBiTmO_4_ could be enhanced, according to the aforementioned studies. While the M’–O–M’ bond angle was close to 180°, the locomotivity of the photoinduced current carrier was powerful; accordingly, a better photocatalytic activity of ZnBiTmO_4_ could be achieved. According to the centrical oxygen atom, the Bi–O–Bi bond angle was 137.369°, meaning that ZnBiTmO_4_ had a better catalytic activity [26].

**Figure 2:**
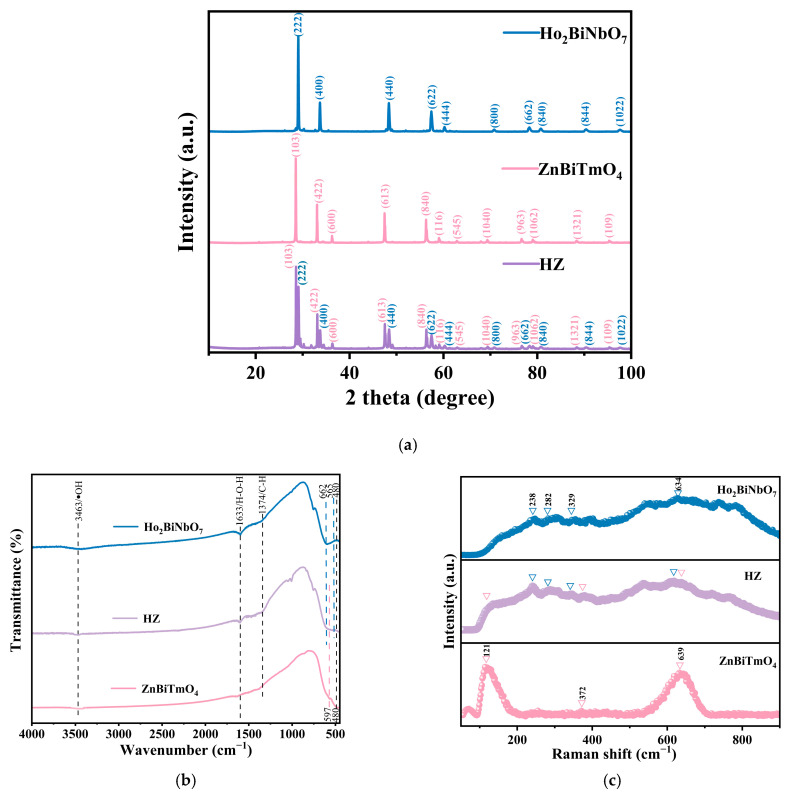
(**a**) XRD plots, (**b**) FTIR spectrograms, and (**c**) Raman spectrograms of Ho_2_BiNbO_7_, ZnBiTmO_4_, and HZ.

Figure 2a presents the XRD patterns of Ho_2_BiNbO_7_, ZnBiTmO_4_, and HZ. It could be seen in Figure 2a that the total diffractive peaks and crystal planes from the XRD patterns of HZ originated from the single-phase Ho_2_BiNbO_7_ or single-phase ZnBiTmO_4_, which verified the preparation of HZ.

In order to understand the surface characteristics, chemical bonds, and functional groups of the prepared catalysts, Fourier transform infrared spectroscopy (FTIR) analysis was conducted. Figure 2b exhibits the FTIR spectrograms of Ho_2_BiNbO_7_, ZnBiTmO_4_, and HZ. As shown in Figure 2b, the vibrational peak at 662 cm^−1^ proved the oscillation of the Nb-O bond [27]. The peaks at 565 cm^−1^ or 480 cm^−1^ were the oscillation of the Ho–O bond or the Bi–O bond [28]. The vibrational peaks observed at clear wavenumbers indicated the successful preparation of the Ho_2_BiNbO_7_ specimen. The peak at 597 cm^−1^ or 480 cm^−1^ was the stretched oscillation of the Tm–O bond or the Bi–O bond [29,30]. The vibrational peaks observed at clear wavenumbers indicated the successful preparation of the ZnBiTmO_4_ specimen. In Figure 2b, the FTIR spectrum of HZ has vibrational bands that remain with Ho_2_BiNbO_7_ and ZnBiTmO_4_. These results further confirmed the successful preparation of the HZ sample. Moreover, the peak at 3463 cm^−1^ represented the stretched oscillation of the hydroxyl group, which could derive from adsorption water or crystal water [31]. The adsorptive band at 1633 cm^−1^ proved the water origin and represented the stretched oscillation of the H–O–H structure. Additionally, the peak at 1374 cm^−1^ was attributed to the symmetric bending model of the C–H bond [32].

Ho_2_BiNbO_7_, ZnBiTmO_4_ and HZ were experimentally investigated using a Raman spectrometer to understand the oscillatory characteristics and crocosmic structural characteristics of Ho_2_BiNbO_7_, ZnBiTmO_4_ and HZ. Figure 2c reveals the Raman spectra of Ho_2_BiNbO_7_, ZnBiTmO_4_ and HZ. On the Raman spectrogram of Ho_2_BiNbO_7_, a peak at 329 cm^−1^ was detected and corresponded to the tensile vibrational model of the Ho–O band [33]. The peak at 238 cm^−1^ could be attributed to the bending oscillation of the Nb–O–Nb bond, which belonged to Ho_2_BiNbO_7._ The peak at 282 cm^−1^ originated from the oscillation of the Bi-O bond; meanwhile, the mode was presented as E1g. The peak at 634 cm^−1^ originated from the other vibrational mode of the Bi–O bond [27]. In the Raman spectrum of Ho_2_BiNbO_7_, the distinctive characteristic peaks at 329 cm^−1^, 238 cm^−1^, 282 cm^−1^ and 634 cm^−1^ confirmed the successful preparation of Ho_2_BiNbO_7_. In the Raman spectrogram of ZnBiTmO_4_, peaks at 372 cm^−1^, 121 cm^−1^ or 639 cm^−1^ might be detected. The above vibrational peaks indicated the successful preparation of ZnBiTmO_4_. The peaks at 121 cm^−1^ and 639 cm^−1^ could be attributed to the vibration of the Bi–O bonds which belonged to ZnBiTmO_4._ The peak at 372 cm^−1^ originated from the A_g_ vibration mode or the combination mode of A_g_ and F_g_ of the Tm–O band [34]. As a whole, the distinct peaks observed in the Raman spectrum of HZ included 329 cm^−1^, 238 cm^−1^, 282 cm^−1^, 634 cm^−1^, 372 cm^−1^, 121 cm^−1^ and 639 cm^−1^. In the Raman spectrum of HZ, the peak vibrations of Ho_2_BiNbO_7_ and ZnBiTmO_4_ could be clearly observed. In combination with the experimental results of FTIR, the Raman spectral results verified the successful preparation of a heterostructure between the single-phase Ho_2_BiNbO_7_ and the single-phase ZnBiTmO_4_, which were prepared in this study.

**Figure 3:**
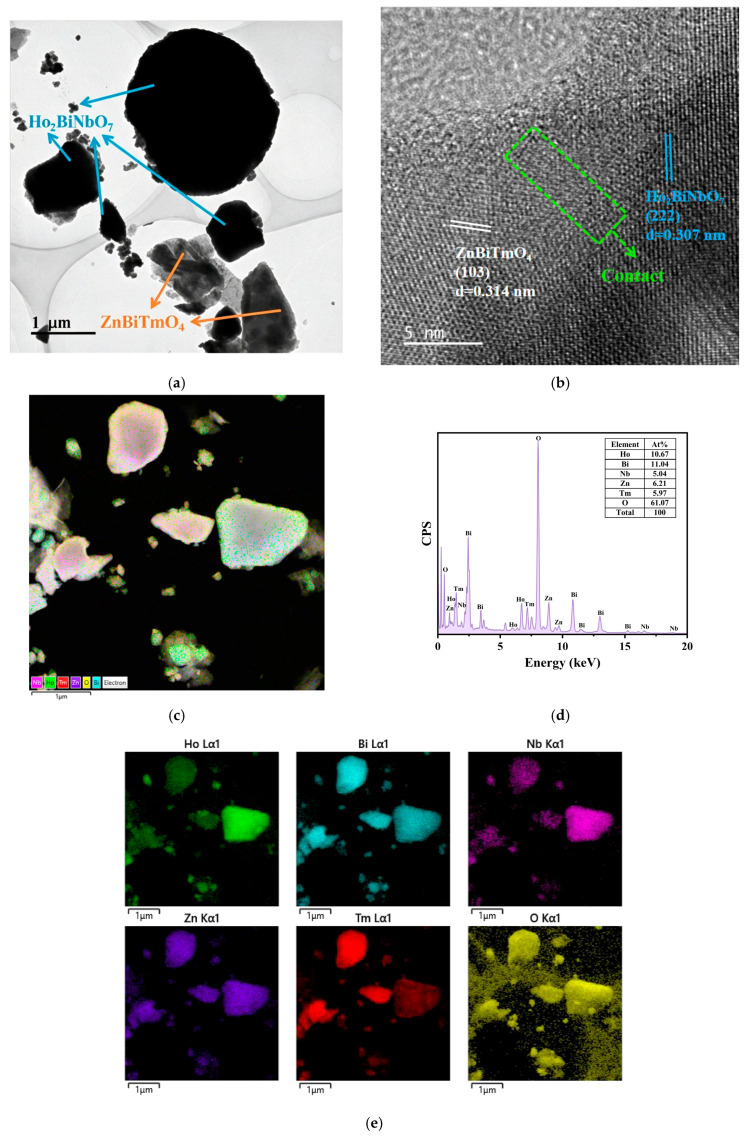
(**a**) TEM, (**b**) HRTEM, (**c**) layered EDS, (**d**) EDS composition profiles, and (**e**) EDS elementary mapping images of HZ.

Transmission electron microscope (TEM) and energy dispersive X-ray spectroscopy (EDS) were utilized to analyze the microscopic structure and elementary constituents of the HZ specimen. Figure 3a exhibits the microstructural morphology of the HZ specimen. It could be observed from Figure 3a that the large-sized grains belonged to Ho_2_BiNbO_7_, with a mean diameter of 1380 nm, and concurrently, the small-sized grains in a white color were ZnBiTmO_4_, with a mean diameter of 890 nm. It could also be found from Figure 3a that the single-phase Ho_2_BiNbO_7_ and the single-phase ZnBiTmO_4_ were equally distributed within the HZ photocatalyst. Figure 3b and Figure S4 present the high-resolution stripe images of HZ. The link between Ho_2_BiNbO_7_ and ZnBiTmO_4_ was obtained, facilitating efficient charge transport between Ho_2_BiNbO_7_ and ZnBiTmO_4_. Moreover, according to Figure 3b, the lattice stripe with an interplanar distance of 0.307 nm or 0.314 nm was found in the crystal plane indices of (222) for the cubic system Ho_2_BiNbO_7_ or the crystal plane indices of (103) for the tetragonal system ZnBiTmO_4_, respectively.

Figure 3c–e exhibit EDS element mapping images of HZ. Ho, Bi, Nb, Zn, Tm, and O elements had a regular distribution within the HZ specimen; meanwhile, other elements could not be measured. This phenomenon proved that there was a single-phase Ho_2_BiNbO_7_ and a single-phase ZnBiTmO_4_ within HZ without other impure phases. The EDS spectrogram in Figure 3d clarified that Ho, Bi, Nb, Zn, Tm and O elements were distributed in HZ according to the atomic ratio. Results of the TEM spectrum and the EDS spectrum for HZ revealed that the resultant HZ specimen had a single-phase Ho_2_BiNbO_7_ and a single-phase ZnBiTmO_4_, while the molar ratio of Ho_2_BiNbO_7_ and ZnBiTmO_4_ was around 1:1.

During the prepared process of HZ, Ho_2_BiNbO_7_ and ZnBiTmO_4_ did not undergo the chemical variation that was needed to generate a novel single-phase substance. In addition, in combination with the results from the XRD plots, the FTIR spectra and the Raman spectrograms, the high-resolution stripe estimation results and the EDS element mapping results clarified both the possibility of the resultant method and the successful preparation of a pure HZ specimen.

Figure S5 shows the nitrogen adsorption–desorption processes of Ho_2_BiNbO_7_, ZnBiTmO_4_ and HZ. Each sample was measured three times, and the average value for the BET surface area of Ho_2_BiNbO_7_, ZnBiTmO_4_ or HZ with standard deviations was reported. According to Figure S5, the specific surface area of HZ was 6.944 m^2^/g, that of Ho_2_BiNbO_7_ was 4.387 m^2^/g, and that of ZnBiTmO_4_ was 4.158 m^2^/g. It was noteworthy that the Brunauer–Emmett–Teller (BET) surface area of HZ was significantly larger than that of Ho_2_BiNbO_7_ or ZnBiTmO_4_, which provided strong evidence for the higher catalytic activity of HZ. The higher specific surface area of HZ was beneficial for supplying more active reaction sites and was considered a crucial factor for improving the photocatalytic efficiency of HZ.

**Figure 4:**
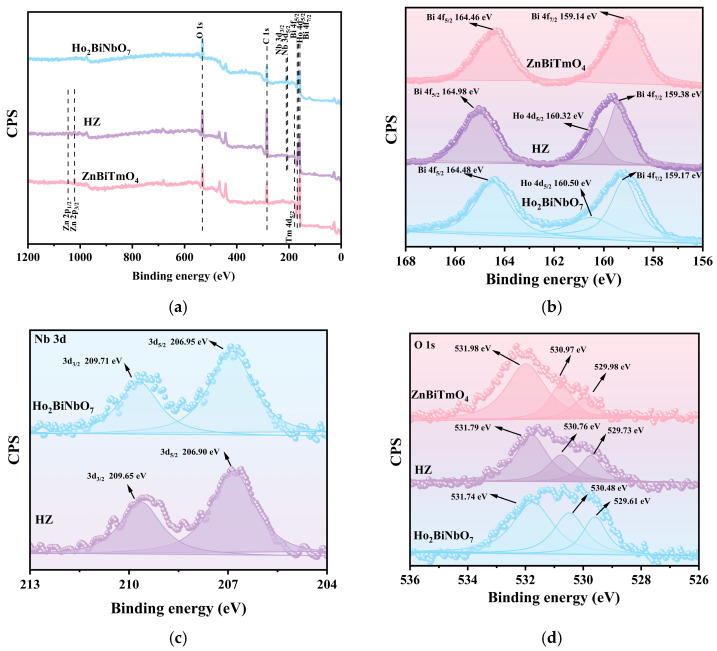

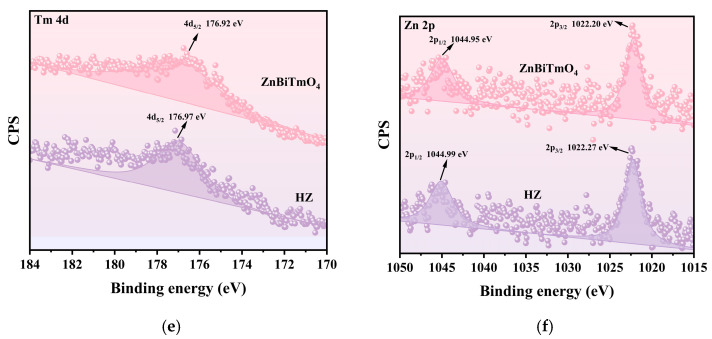
The XPS spectra of prepared Ho_2_BiNbO_7_, ZnBiTmO_4_, and HZ: (**a**) survey spectra; (**b**–**f**) high-resolution XPS spectra of Bi 4f, Nb 3d, O 1s, Tm 4d, and Zn 2p.

In order to determine the specimen element content and the elemental chemical valence, an X-ray photoelectron spectrometer (XPS) was utilized. Figure 4 reveals the XPS spectra of the prepared Ho_2_BiNbO_7_, ZnBiTmO_4_ and HZ. The survey spectrum of HZ shown in Figure 4a verified the presence of Ho, Bi, Nb, Zn, Tm and O elements, indicating that these elements stemmed from Ho_2_BiNbO_7_ and ZnBiTmO_4_. In the Bi 4f orbital shown in Figure 4b, if we consider spin–orbit interaction, which can be divided into spin-up and spin-down, the Bi 4f orbital that remains with ZnBiTmO_4_ may be divided into a couple of peaks involving Bi 4f_5/2_ and Bi 4f_7/2_. Concurrently, the peak of Bi 4f_5/2_ emerged at 164.46 eV, that of Bi 4f_7/2_ emerged at 159.14 eV, that of Bi 4f_5/2_ emerged at 164.48 eV, and that of Bi 4f_7/2_ emerged at 159.17 eV in Ho_2_BiNbO_7_. Concurrently, in HZ, two peaks emerged, at 164.98 eV and 159.38 eV [35]. As for the Ho 4d orbital in Figure 4b, the peak of Ho 4d_5/2_ belonging to Ho_2_BiNbO_7_ is at 160.50 eV. As for HZ, the peak of Ho 4d_5/2_ is at 160.32 eV [36]. For the Nb 3d orbital in Figure 4c, the binding energy position of the Nb 3d_5/2_ orbital belonging to Ho_2_BiNbO_7_ is at 206.95 eV, while the binding energy position of the Nb 3d_3/2_ orbital is at 209.70 eV. In HZ, the binding energy position of the Nb 3d_5/2_ orbital is at 206.90 eV, while the binding energy position of the Nb 3d_5/2_ orbital is at 209.65 eV. For the O 1s orbital in Figure 4d, the peaks of O 1s remaining with Ho_2_BiNbO_7_ were 531.74 eV, 530.48 eV, and 529.60 eV. In ZnBiTmO_4_, the peaks of O 1s were at 531.98 eV, 530.97 eV, and 529.98 eV. As for HZ, the peaks of O 1s were at 531.79 eV, 530.76 eV, and 529.74 eV. For the Tm 4d orbital in Figure 4e, in ZnBiTmO_4_, the peak of Tm 4d_5/2_ was at 176.92 eV. In HZ, the peak of Tm 4d_5/2_ was at 176.97eV. The Zn 2p orbital, which was exhibited in Figure 4f, was split into Zn 2p_1/2_ orbital and Zn 2p_3/2_ orbital, which were considered as spin–orbit coupling; the peak of Zn 2p_1/2_ was at 1044.95 eV, while the peak of Zn 2p_3/2_ was at 1022.20 eV. In HZ, the peaks of Zn 2p_1/2_ and Zn 2p_3/2_, 1044.99 eV and 1022.27 eV, generated a tiny shift [37].

For the Ho 4d orbital and Nb 3d orbital, the binding energy localities from HZ had a small negative shift compared to that of Ho_2_BiNbO_7_. For the Zn 2p orbital and Tm 4d orbital, the binding energy localities in HZ had a small positive movement. This phenomenon formed a Z-type heterojunction, triggering the consumption of electronic density in ZnBiTmO_4_ and concurrent electronic accumulation in Ho_2_BiNbO_7_. This orbit binding energy shift was attributed to the successful construction of a Z-scheme heterojunction, which induced electron density depletion in ZnBiTmO_4_ and concurrent electron accumulation in Ho_2_BiNbO_7_ [27]. Meanwhile, these findings revealed composite electronic interaction in the intramural energy band structure of the HZ photocatalyst; accordingly, the functional photocatalytic activity of HZ could be enhanced [38]. The XPS spectra of the fresh HZ catalyst and the HZ sample that was collected after five consecutive photocatalytic degradation cycles are shown in Figure S6. The extra peaks were not detected in the spectra of the HZ sample that was collected after five consecutive photocatalytic degradation cycles, indicating that noticeable chemical change did not occur. These findings certified the excellent stability of the HZ catalyst under sustained photocatalytic activity.

In the XPS analysis results of Ho_2_BiNbO_7_, ZnBiTmO_4_ and HZ, an impure phase was not observed. The XPS analysis results supplied crucial evidence for exploiting a Z-type heterostructure in the HZ photocatalyst. In combination with the TEM results, the HZ photocatalyst verified the powerful chemical interaction between Ho_2_BiNbO_7_ and ZnBiTmO_4_. Furthermore, these results complemented and strengthened observations deriving from other characterization techniques, such as X-ray diffraction, Fourier transform infrared spectroscopy, Raman optical spectroscopy, transmission electron microscope, high-resolution transmission electron microscope, and energy dispersive X-ray optical spectroscopy.

**Figure 5:**
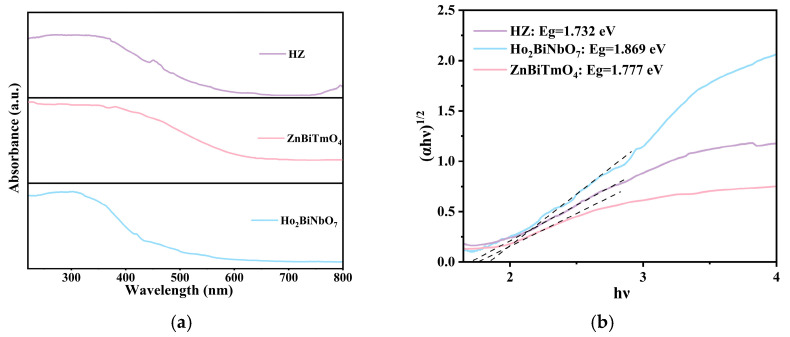
(**a**) The ultraviolet and visible absorption spectra of Ho_2_BiNbO_7_, ZnBiTmO_4_, and HZ; (**b**) corresponding plots of (*αhν*)^1/2^ and *hν* obtained according to Figure 5a and formula 2 for Ho_2_BiNbO_7_, ZnBiTmO_4_, and HZ.

In order to study the optic responsive characteristic and the band structure of the prepared specimens, the ultraviolet and visible absorption spectra of Ho_2_BiNbO_7_, ZnBiTmO_4_ and HZ were tested. Figure 5a exhibits the ultraviolet and visible absorption spectra of Ho_2_BiNbO_7_, ZnBiTmO_4_, and HZ. It could be seen in Figure 5a that Ho_2_BiNbO_7_, ZnBiTmO_4_, and HZ displayed clear absorption peaks from 400 nm to 700 nm, clarifying that Ho_2_BiNbO_7_, ZnBiTmO_4_ and HZ were responsive to VILIIR. The bandgap width of *Eg* was confirmed by the crossing point that appeared between the *hυ* shaft axle and the direct line, which originated from the rectilinear speculation of the absorbing factor. The corresponding curved line was described as the Kubelka–Munk formula [39].

(1) $$F_{KM}[R_{d}(h\nu )]=\frac{{\left[1-R_{d}(h\nu )\right]}^{2}}{2R_{d}(h\nu )}=\frac{\alpha h\nu }{S}$$

This mathematical equation assigned the scattering eigenvalue to *S*, the diffuse reflectance modulus to *R_d_* and the radiation absorption modulus to *α*.

For different gamma-ray transition mechanisms, the incident photonic energy and the bandgap width *Eg* of the semiconductor catalyst had the following ordinary relationship [40].

(2) $${\left(\alpha h\nu \right)}^{n}=K(h\upsilon -Eg)$$

Here, *α* represents the absorbing factor, *h* represents Planck’s constant, *υ* represents the frequency, *K* represents the unchangeable constant, and *Eg* represents the bandgap width. In this formula, *n* represents the characteristic of the transition in a semiconducting catalyst. *n* can be estimated by the following procedures: (i) the effect of *ln*(*αhυ*) on *ln*(*hυ − Eg*) may be revealed by presuming a proximate magnitude of *Eg*, or (ii) the exponent *n* was deduced by analyzing the slope of the linear region in the plot of *ln*(*αhυ*) versus *ln*(*hυ − Eg*). Using these methods, the *n* value of Ho_2_BiNbO_7_, ZnBiTmO_4_ or HZ may be confirmed. When the *n* value was equivalent to 2 or 2/3, it presented a direct lambda or direct transition. When the *n* value was equivalent to 1/2 or 1/3, it presented an indirect lambda or indirect transition. By using the above methods, the *n* value for Ho_2_BiNbO_7_, ZnBiTmO_4_ or HZ was determined to be 1/2, indicating that Ho_2_BiNbO_7_, ZnBiTmO_4_ or HZ possessed an indirect bandgap.

In Figure 5b, a plot shows the effect of (*αhν*)^1/2^ on *hν* for Ho_2_BiNbO_7_, ZnBiTmO_4_ and HZ. The vertical axis is plotted as (*αhν*)^1/2^, which represents the transition mode of the photocatalysts. The bandgap width of Ho_2_BiNbO_7_, ZnBiTmO_4_ or HZ derived from the intercept. Using Figure 5b, the bandgap energy value of Ho_2_BiNbO_7_, ZnBiTmO_4_ or HZ might be estimated. We found that Ho_2_BiNbO_7_ had an indirect bandgap with a width of 1.869 eV, ZnBiTmO_4_ had an indirect bandgap with a width of 1.777 eV, and HZ had an indirect bandgap with a width of 1.732 eV. It could be deduced from Figure 5a and Figure 5b that the bandgap width of HZ was narrower in comparison with that of Ho_2_BiNbO_7_ or ZnBiTmO_4_. It could be concluded from Figure 5b that the bandgap width of HZ was closely related to those of Ho_2_BiNbO_7_ and ZnBiTmO_4_, which displayed different transition characters.

These results revealed that under the identical energy of VILIIR, the HZ photocatalyst might immensely decrease the energy barrier for electronic transition, so more electrons might be facilitated for transiting from the valence band of HZ to the conductance band of HZ. Therefore, the production of photogenerated electron–hole pairs might be enhanced, and a strong catalytic activity of HZ might be achieved.

### 3.2. Catalytic Activity Examination

#### 3.2.1. Catalytic Degradation of Brilliant Green

**Figure 6:**
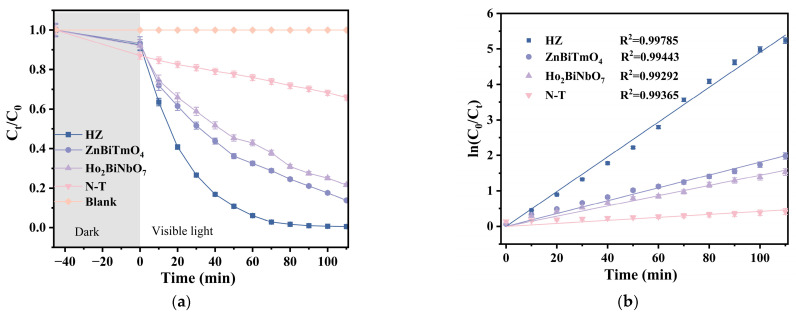

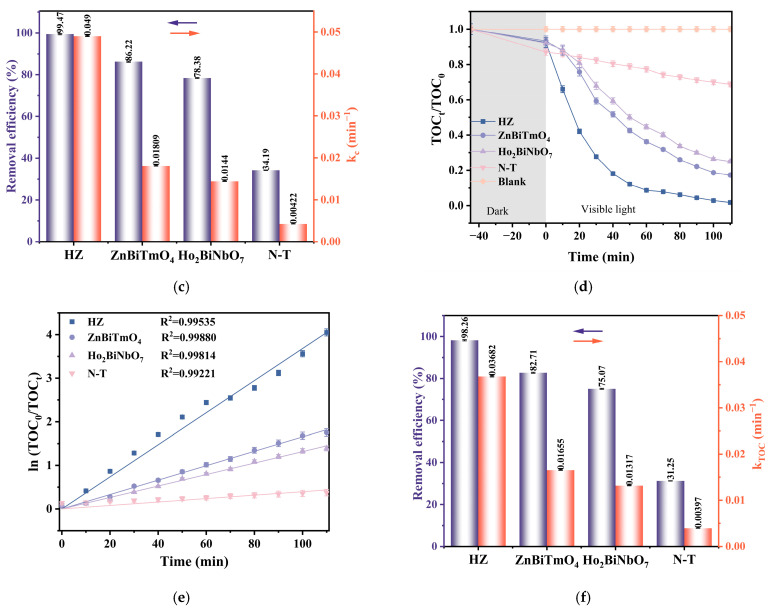
(**a**) The degradation curves of BLG, (**b**) kinetic curves for BLG concentration variation, (**c**) degradation efficiencies and kinetic constants for the BLG concentration, (**d**) mineralized curves for removing TOC concentration, (**e**) kinetic curves for TOC concentration variation, and (**f**) mineralization efficiencies and kinetic constants for TOC concentration with Ho_2_BiNbO_7_, ZnBiTmO_4_, HZ or N-T as the photocatalyst under visible light irradiation.

Figure 6a exhibits degradation curves of BLG with Ho_2_BiNbO_7_, ZnBiTmO_4_, HZ or N-T as the photocatalyst under VILIIR. Prior to irradiation, the suspension was stirred in the dark for 45 min to reach adsorption–desorption equilibrium. The N-T catalyst was prepared and investigated in the industrial wastewater treatment field; N-T is an acknowledged visible light-responsive catalyst, widely used in the catalytical reagent market. The N-T catalyst might be employed as a contrapositive catalyst to evaluate the difference in photocatalytic activity compared with other catalysts. The N-T catalyst exhibited the diagnostic structure of the octahedrite phase for TiO_2_ (JCPDS No. 21-1272), and the bandgap width of the N-T catalyst was detected as 2.92 eV. It could be discovered from Figure 6a that the degradation rate of BLG was 78.38%, 86.22%, 99.47% or 34.19% with Ho_2_BiNbO_7_, ZnBiTmO_4_, HZ or N-T as the photocatalyst after VILIIR for 110 min, indicating that HZ had a higher catalytic activity than Ho_2_BiNbO_7_, ZnBiTmO_4_ or N-T. According to the contrastive experiments, it was found that the BLG concentration did not vary significantly without increasing other photocatalysts. These results clarified that the fast removal rate of BLG was produced by the high photocatalytic activity of HZ, while the photolytic reactivity characteristic was not presented.

Figure 6b displays the kinetic curves for the BLG concentration variation with Ho_2_BiNbO_7_, ZnBiTmO_4_, HZ or N-T as the photocatalyst under VILIIR. The curves from the influence of ln(*C*_0_/*C_t_*) on the VILIIR time obey a first-order dynamics model. The first-order kinetic constants can be computed with the following formula:

(3) $$\ln(C_{0}/C_{t})=k_{C}t$$

where *C_t_* represents the BLG concentration at the intermediate VILIIR time, *C*_0_ represents the BLG concentration at the starting VILIIR time, *k_C_* represents the kinetic constant, and *t* represents the time of VILIIR. Figure 6c illustrates the removal efficiencies and the first-order kinetic constants for the BLG concentration, with Ho_2_BiNbO_7_, ZnBiTmO_4_, HZ or N-T as the photocatalyst under VILIIR. As shown in Figure 6c, the degradation rate of BLG with Ho_2_BiNbO_7_, ZnBiTmO_4_, HZ or N-T as the catalyst followed the descending order of HZ > ZnBiTmO_4_ > Ho_2_BiNbO_7_ > N-T. The degradation rate of BLG when employing HZ as the catalyst was enhanced by 1.27, 1.15, or 2.91 times compared to that when employing Ho_2_BiNbO_7_, ZnBiTmO_4_, or N-T as the catalyst. Moreover, the kinetic constant, which originated from the effect of the BLG concentration and VILIIR time when employing HZ, was enhanced by 3.40, 2.71, or 11.61 times compared to that when using Ho_2_BiNbO_7_, ZnBiTmO_4_, or N-T. Figure S7a exhibits the degradation curves of the five experimental rotations for degrading BLG with HZ as a catalyst under VILIIR. Figure S7b illustrates the kinetic curves of quintic experimental cycles for degrading BLG with HZ as the catalyst under VILIIR. Figure S7c displays the degradation rates and kinetic constants of quintic cyclic experiments for degrading BLG by employing HZ under VILIIR. Figure S7d reveals the mineralized curves for removing total organic carbon (TOC) concentration (TOC-C) after quintic cyclic degradation detection for degrading BLG with HZ as the photocatalyst under VILIIR. Figure S7e indicates the kinetic curves for TOC-C variation after quintic cyclic degradation tests for degrading BLG with HZ as the catalyst under VILIIR. Figure S7f illustrates the mineralization rates and kinetic constants of quintic cyclic experiments for removing the TOC-C when using HZ under VILIIR. As shown in Figure S7a–c, after quintic cyclic experiments, the degradation rate of BLG when employing HZ still remained at 94.03%, showing that the removal rate of the BLG concentration only decreased by 5.44% when using HZ under VILIIR after quintic cyclic experiments. These experimental results revealed that the HZ photocatalyst possessed excellent stabilization and reusability and a high photocatalytic activity.

The photonic efficiency was estimated using Formula (4) [41,42].

(4) $$\phi =R/I_{0}\times 100\%$$

Here, ϕ represents the photonic efficiency (%), *R* represents the degradation rate of BLG (Mol·L^−1^·s^−1^), and $I_{0}$ represents the light quantum flux (Einstein·L^−1^·s^−1^). The incident light intensity $I_{0}$ which was detected by a radiometer (Model FZ-A, Beijing, China), was found to be 4.76 × 10^−6^ Einstein·L^−1^·s^−1^ under VILIIR.

The photonic efficiency was computed to be 0.1013%, 0.0878%, 0.0798% or 0.0348% with HZ, ZnBiTmO_4_, Ho_2_BiNbO_7_ or N-T as the photocatalyst under VILIIR. The photonic efficiency when employing HZ was the highest when compared with that with ZnBiTmO_4_, Ho_2_BiNbO_7_ or N-T as the photocatalyst, indicating that HZ had the highest photocatalytic activity among ZnBiTmO_4_, Ho_2_BiNbO_7_ and N-T.

Figure 6d displays the mineralized curves for removing TOC-C in the process of degrading BLG in wastewater with Ho_2_BiNbO_7_, ZnBiTmO_4_, HZ or N-T as the photocatalyst under VILIIR. As shown in Figure 6d, Ho_2_BiNbO_7_, ZnBiTmO_4_ or HZ obtained a high mineralization rate for removing TOC-C; meanwhile, the mineralized results of TOC-C with Ho_2_BiNbO_7_, ZnBiTmO_4_, HZ or N-T as the photocatalyst under VILIIR were consistent with the above degradation results of BLG. It could be found from Figure 6d that the removal rate of TOC-C was 75.07%, 82.71%, 98.26% or 31.25% with Ho_2_BiNbO_7_, ZnBiTmO_4_, HZ or N-T as the photocatalyst after VILIIR for 110 min, indicating that HZ had a higher mineralization efficiency than Ho_2_BiNbO_7_, ZnBiTmO_4_ or N-T. Figure 6e reveals the kinetic curves caused by TOC-C variation with Ho_2_BiNbO_7_, ZnBiTmO_4_, HZ or N-T as the photocatalyst under VILIIR. It could be seen from Figure 6e that the kinetic curves fit a first-order kinetic model, and thus the first-order kinetic constants during the mineralized course of TOC-C could be estimated. The first-order kinetic constant from the effect of ln(*TOC*_0_/*TOC_t_*) on the VILIIR time was estimated by employing the following formula:

(5) $$\ln(TOC_{0}/TOC_{t})=k_{TOC}t$$

where *TOC_t_* represents TOC-C at the intermediate VILIIR time, and *TOC*_0_ represents TOC-C at the starting VILIIR time. In order to clarify the mineralization rate of TOC-C, Figure 6f illustrates the mineralization rate for removing TOC-C and kinetic constants with Ho_2_BiNbO_7_, ZnBiTmO_4_, HZ or N-T as the photocatalyst under VILIIR. As revealed in Figure 6f, the mineralization rates for removing TOC-C when using different catalysts followed this sequence: HZ > ZnBiTmO_4_ > Ho_2_BiNbO_7_ > N-T. The analysis results indicated that the HZ had the best mineralization rate for removing TOC-C in the degradation of BLG, surpassing Ho_2_BiNbO_7_, ZnBiTmO_4_ and N-T. The mineralization rate for removing TOC-C when employing the HZ was 1.19 times higher than that when employing ZnBiTmO_4_, 1.31 times higher than that when employing Ho_2_BiNbO_7_, and 3.14 times higher than that when employing N-T. Concurrently, the kinetic constant *k_TOC_* which was obtained from the curve of the effect of TOC-C on the VILIIR time when employing the HZ as the catalyst increased by 2.22 times, 2.80 times, or 9.27 times when compared with that when using ZnBiTmO_4_, Ho_2_BiNbO_7_, or N-T as the catalyst. In Figure S7d–f, the mineralization rate of TOC-C when employing HZ remained at 93.01% after quintic cyclic experiments, indicating that the mineralization rate of TOC-C decreased by only 5.25% with HZ as the catalyst after quintic cyclic experiments. This proved that the resultant HZ had higher stability and satisfactory reusability in terms of catalytic activity. The blank control experiment without any photocatalyst was accomplished for evaluating the effect of photolysis. The mineralization rate of TOC-C in this case was negligible after VILIIR of 110 min.

An atomic absorption spectrometer was employed to detect the leaching elements after photocatalytic treatment within the water solution containing the BLG. Accordingly, we did not detect the toxic leaching elements such as holmium element, bismuth element, niobium element, zinc element, or thulium element after photocatalytic treatment of the BLG by utilizing Ho_2_BiNbO_7_, ZnBiTmO_4_ or HZ. Thus, the ultimate products in this experiment were innocuous to the water environment.

In order to provide a more rigorous demonstration of the novelty and superiority of the prepared HZ photocatalyst, the photocatalytic performance of the HZ photocatalyst was systematically compared with that of recently reported pyrochlore photocatalysts, spinel photocatalysts, and Z-scheme heterojunction photocatalysts (Table S4) [10,43,44]. The superior photocatalytic performance of the HZ photocatalyst could be attributed to the synergistic effects of (i) the matched band structures of Ho_2_BiNbO_7_ and ZnBiTmO_4_ which could promote the Z-scheme charge transfer, (ii) the formation of a robust interfacial electric field, which could facilitate the charge separation, and (iii) the efficient separation of the photogenerated current carrier.

In order to lessen the potential influence of dye photosensitization, colorless phenol was employed as a model substrate to assess the photocatalytic activity of Ho_2_BiNbO_7_, ZnBiTmO_4_ and HZ, as presented in Figure S8. It could be found from Figure S8 that Ho_2_BiNbO_7_, ZnBiTmO_4_ and HZ exhibited exceptionally high photocatalytic activity during the degradation process of phenol under the condition of VILIIR. Specifically, the removal rate of phenol was 90.00%, and the mineralization rate for removing TOC-C was 88.67% when employing HZ after VILIIR for 110 min. According to Figure S8, HZ had the highest removal efficiency and mineralization efficiency during the degradation process of phenol, surpassing Ho_2_BiNbO_7_ and ZnBiTmO_4_. These findings demonstrated that photosensitization was not the dominant factor during the photocatalytic degradation process of BLG when using Ho_2_BiNbO_7_, ZnBiTmO_4_ or HZ; instead, the efficient degradation of BLG originated from the intrinsic photocatalytic activity of Ho_2_BiNbO_7_, ZnBiTmO_4_ or HZ. This provided further evidence for the proposed Z-scheme heterojunction mechanism.

**Figure 7:**
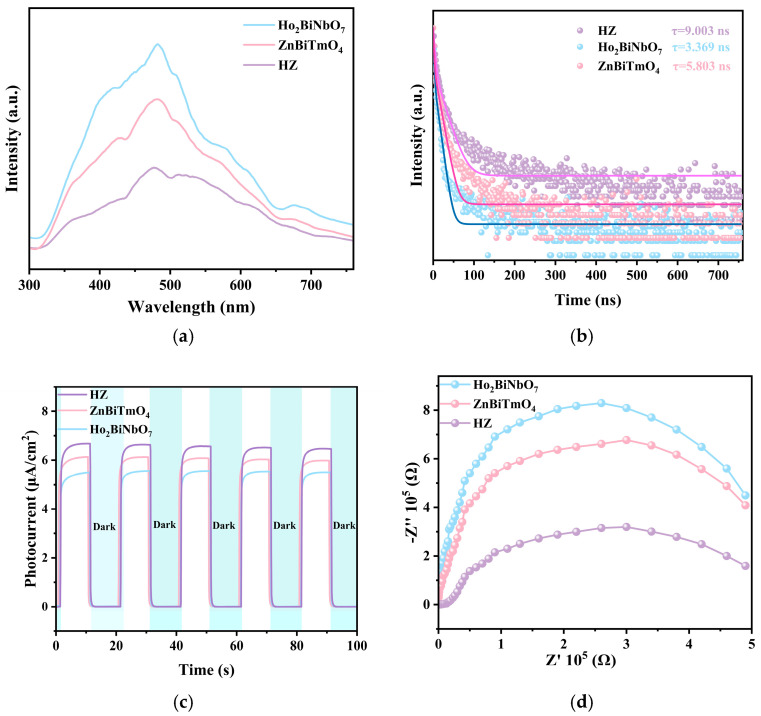
(**a**) PL spectra, (**b**) TRPLC spectra, (**c**) transient photocurrent curves, and (**d**) electrochemical impedance spectroscopy (EIS) plots for Ho_2_BiNbO_7_, ZnBiTmO_4_, and HZ.

The photoluminescence (PL) spectra of Ho_2_BiNbO_7_, ZnBiTmO_4_, and HZ are shown in Figure 7a [45]. Here, the PL intensity of the HZ specimen is the lowest in comparison with those of Ho_2_BiNbO_7_ and ZnBiTmO_4_. Furthermore, it could be observed from Figure 7a that photogenerated electrons and photogenerated holes produced in the HZ specimen had the lowest recombination rate in comparison with those in the Ho_2_BiNbO_7_ specimen and the ZnBiTmO_4_ specimen. Therefore, HZ exhibited the highest photocatalytic activity in comparison with Ho_2_BiNbO_7_ or ZnBiTmO_4_. These results further revealed that HZ had a strong ability to degrade BLG when compared with Ho_2_BiNbO_7_ or ZnBiTmO_4_. This finding was consistent with our previous analytic results.

Time-resolved photoluminescence (TRPLC) spectrograms of Ho_2_BiNbO_7_, ZnBiTmO_4_ and HZ are shown in Figure 7b [46]. According to Figure 7b and the following formula, the fluorometric lifetime (*τ_ανe_*) of HZ could be estimated to be 9.003 ns. *τ*_1_ and *τ*_2_ are homologous parameters of the fluorometric lifetime, while *A*_1_ and *A*_2_ are normal simulated parameters [47].

(6) $$\tau_{ave}=(A_{1}{\tau_{1}}^{2}+A_{2}{\tau_{2}}^{2})/(A_{1}\tau_{1}+A_{2}\tau_{2})$$

The fluorometric lifetime of Ho_2_BiNbO_7_ or ZnBiTmO_4_ was 3.369 ns or 5.803 ns, respectively. This result showed that photogenerated electrons and photogenerated holes which were produced on the surface of the HZ grain were harder to recombine; therefore, HZ obtained higher photocatalytic activity in comparison with Ho_2_BiNbO_7_ or ZnBiTmO_4_. These results were consistent with the experiments on the PL spectra of Ho_2_BiNbO_7_, ZnBiTmO_4_, and HZ, indicating that the direct Z-type HZ photocatalyst had the highest catalytic properties in comparison with the single-phase Ho_2_BiNbO_7_ and the single-phase ZnBiTmO_4_.

In order to analyze the separation efficiency and diffusion efficiency of the photogenerated electrons and the photogenerated holes which are produced on the surface of the catalyst grain, transient photocurrent intensity variation experimentation is performed with Ho_2_BiNbO_7_, ZnBiTmO_4_, or HZ as the resultant membrane specimen, and the experimental results are shown in Figure 7c [48]. According to Figure 7c, the transient photocurrent intensity of the HZ specimen was higher compared to that of the Ho_2_BiNbO_7_ specimen and the ZnBiTmO_4_ specimen. The results indicated that the HZ specimen had the highest usage efficiency of light energy and the maximum separation efficiency of the photogenerated electrons and photogenerated holes with the incident light irradiation.

Ho_2_BiNbO_7_, ZnBiTmO_4_, and HZ displayed different charge transfer characteristics, according to the Nyquist plot of electrochemical impedance spectroscopy (EIS) in Figure 7d [49]. The semicircle diameter for the HZ photocatalyst was considerably less than that for the Ho_2_BiNbO_7_ photocatalyst or the ZnBiTmO_4_ photocatalyst, showing that the HZ photocatalyst possessed a lower charge transfer impedance. These results showed that the photogenerated electron–hole pairs generated on the surface of the HZ grain had a higher separation efficiency than that of the Ho_2_BiNbO_7_ grain or the ZnBiTmO_4_ grain. The descriptive results verified the strong catalytic activity of the HZ photocatalyst. These conclusions were mutually proven by the photocurrent intensity variation test results and the PL spectrum analysis results, confirming that the HZ photocatalyst possessed excellent photocatalytic properties and thus represented a high-efficiency photocatalytic specimen.

**Figure 8:**
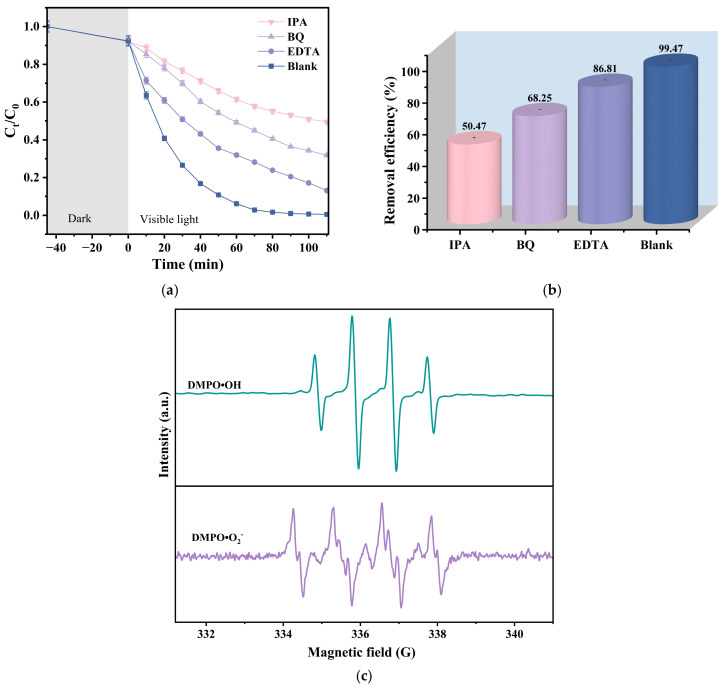
Effect of different radical scavengers on (**a**) the BLG concentration, and (**b**) the removal efficiency of the BLG concentration by employing HZ under visible light irradiation; (**c**) the EPR spectra for DMPO•O_2_^–^ and DMPO•OH by employing HZ.

In order to study the contribution of active radicals from HZ during the catalytic degradation process of BLG, radicals trapping experiments were performed. The effects of different active radicals were estimated by adding different radicals scavengers. Isopropanol (IPA) was employed to capture hydroxyl radicals (•OH), while benzoquinone (BQ) was used to capture superoxide anions (•O_2_^−^), and ethylenediaminetetraacetic acid (EDTA) was employed to capture the photogenerated holes (h^+^). The experimental results are shown in Figure 8a,b [50]. Without the addition of EDTA, BQ or IPA, the degradation rate of BLG was 99.47%. Nevertheless, the degradation rate of BLG was reduced to 86.81%, 68.25% or 50.47% when EDTA, BQ or IPA was used as a scavenging agent. These results clarified that the oxidative ability of •OH was far higher than that of •O_2_^−^ or h^+^ in the catalytic degradation of BLG with HZ as the photocatalyst under VILIIR [51].

In order to provide direct spectral evidence for the generation of these reactive species, the electron paramagnetic resonance (EPR) experiment was completed with DMPO (5,5-dimethy-l-pyrroline N-oxide) as the trapped agent. Figure 8c reveals the EPR spectra for DMPO•O_2_^−^ and DMPO•OH with HZ as the photocatalyst. According to Figure 8c, the reactive radicals •OH was generated and measured in the catalytic degradation of BLG by employing HZ under VILIIR. It could be observed that the intensity ratio of four peaks for •OH was 1:2:2:1; accordingly, above 1:2:2:1 represented an inimitable signal that remained with •OH. When dimethyl sulfoxide was utilized as a dispersing agent and DMPO was used as a trapped agent, the active radicals of •O_2_^−^ which was generated during the catalytic degradation of BLG with HZ as the photocatalyst was investigated. We found that the density of •OH was considerably higher than that of •O_2_^−^. These results were consistent with the experimental results which stemmed from radicals clearance experiment. Based on the radicals scavenging experiments and the EPR experiment analysis results, the descending order of the oxidizing capacity for the three radicals was determined as follows: •OH > •O_2_^−^ > h^+^.

#### 3.2.2. Possible Catalytic Degradation Mechanism of BLG

**Figure 9:**
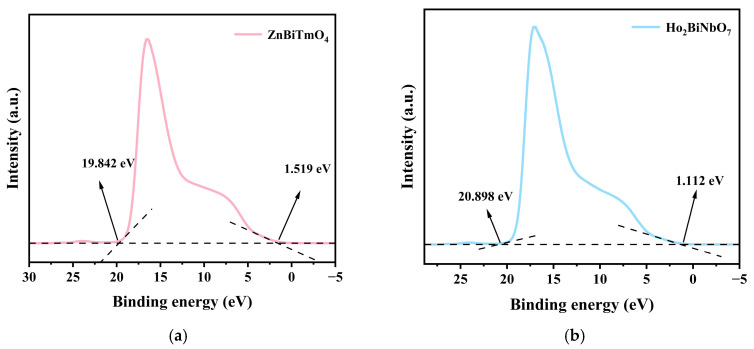
The ultraviolet photoelectron spectrograms of (**a**) ZnBiTmO_4_ and (**b**) Ho_2_BiNbO_7_ (The horizontal dash line represents that the intensity value of the ordinate axis is equal to zero).

In order to determine the valence electron interaction near the Fermi level of the photocatalyst and the ionic potential of the valence band (or occupancy status), the ultraviolet photoelectron spectrogram (UPS) experiment was performed using the Ho_2_BiNbO_7_ specimen or the ZnBiTmO_4_ specimen. Figure 9a and Figure 9b exhibit the UPS spectra of the ZnBiTmO_4_ specimen and the Ho_2_BiNbO_7_ specimen, respectively. The UPS measurements were performed using a He I excitation source with a photon energy of 21.2 eV. When analyzing the UPS spectra of the single-phase Ho_2_BiNbO_7_ photocatalyst and the single-phase ZnBiTmO_4_ photocatalyst by employing the extrapolation method, the onset (Ei) binding energy and the cutoff (Ecutoff) binding energy could be obtained [52]. Both the secondary electron cutoff and the onset were determined by the linear extrapolation method. The values were taken as the intercepts of the linear fit with the *x*-axis; subsequently, one was on the high binding energy side of the secondary electron edge, and the other was on the low binding energy side of the secondary electron edge. Further, the ionic potentials of the valence band positions for Ho_2_BiNbO_7_ and ZnBiTmO_4_ were 1.414 eV and 2.877 eV, respectively. According to the ultraviolet and visible absorption spectra analysis results, the bandgap energies (*Eg*) of Ho_2_BiNbO_7_ and ZnBiTmO_4_ were obtained. Consequently, the electrochemical potentials of the conduction bands for Ho_2_BiNbO_7_ and ZnBiTmO_4_ were computed according to the following formula:

(7) $$E_{VB}=E_{g}+E_{CB}$$

where *Eg* is the bandgap energy of the catalyst, *E_VB_* is the ionic potential of the valence band position for the catalyst, and *E_CB_* is the electrochemical potential of the conduction band position for the catalyst. According to Formula (7), the *E_CB_* for Ho_2_BiNbO_7_ was −0.455 eV, and the *E_CB_* for ZnBiTmO_4_ was 1.100 eV.

The electrochemical potential −0.455 eV of the conduction band for Ho_2_BiNbO_7_ was more negative than the standard potential of O_2_/•O_2_^−^ (−0.33 eV vs. NHE), illustrating that Ho_2_BiNbO_7_ had a stronger reductive capability. These results indicated that the electrons in the conduction band position of Ho_2_BiNbO_7_ were caught by the adsorptive oxygen molecule; accordingly, the oxygen molecule might be readily reduced to for producing •O_2_^−^. The ionic potential 2.877 eV of the valence band for ZnBiTmO_4_ was more positive compared with the standard potential of OH^−^/•OH (2.38 eV vs. NHE), illustrating that ZnBiTmO_4_ possessed a stronger oxidative capability. These results indicated that the photoinduced holes in the valence band position of ZnBiTmO_4_ might oxidize hydroxyl or water for producing •OH. The analytic results were consistent with the experimental results of the EPR testing [53].

**Figure 10:**
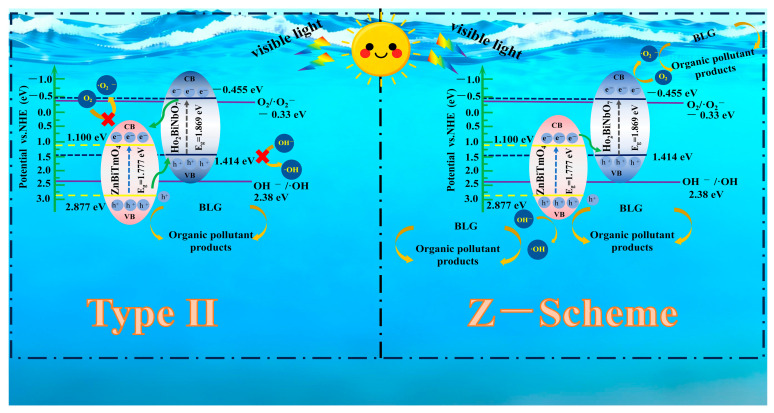
The photodegradation mechanism of BLG with HZ as a photocatalyst.

According to the detailed analyses of the experimental results, the intramural electric charge migration route of the HZ was described. Figure 10 reveals two possible degradation mechanisms of the BLG, direct Z-scheme and Type-II, with HZ as the photocatalyst [54,55]. Based on the comprehensive experimental evidence presented above, a direct Z-scheme charge transfer mechanism was proposed for the HZ composite, rather than the conventional Type-II mechanism. This conclusion was substantiated by the following evidence.

First, as determined by UPS and UV-Vis analyses, the conduction band (CB) potential of Ho_2_BiNbO_7_ (−0.455 eV) was sufficiently more negative than the standard potential of O_2_/•O_2_^−^ (−0.33 eV vs. NHE). The valence band (VB) potential of ZnBiTmO_4_ (2.877 eV) was sufficiently more positive than the standard potential of OH^−^/•OH (2.38 eV vs. NHE). If the Type-II mechanism was dominant, the electrons would accumulate on the CB of ZnBiTmO_4_ (1.100 eV), which is incapable of producing •O_2_^−^. The holes would accumulate on the VB of Ho_2_BiNbO_7_ (1.414 eV), which is incapable of producing •OH. Since the radical trapping and EPR experiments clearly confirmed the generation of both •O_2_^−^ and •OH, the Type-II mechanism could be conclusively ruled out. Second, the XPS binding energy shifts clearly indicated electron density depletion in ZnBiTmO_4_ (positive shift in Zn 2p and Tm 4d) and electron accumulation in Ho_2_BiNbO_7_ (negative shift in Ho 4d and Nb 3d) upon heterojunction formation. This created an internal electric field at the interface, which acted as the driving force for the recombination of electrons from the CB of ZnBiTmO_4_ with the holes from the VB of Ho_2_BiNbO_7_. This mechanism was consistent with the Z-scheme charge migration pathway model. Third, the substantial quenching of photoluminescence intensity, the extended fluorescence lifetime, the increased photocurrent density, and the reduced electrochemical impedance arc radius in the HZ sample confirmed that the Z-scheme charge migration effectively suppressed the recombination of photogenerated electrons and holes, thereby significantly enhancing the photocatalytic activity. Consequently, the direct Z-scheme mechanism not only facilitated the fast separation of the photogenerated electrons and photogenerated holes but also guaranteed the enhancement of the reduction potential and the oxidic potential. The combined evidence from HRTEM observation of the intimate interfacial contact (Figure 3b and Figure S4), the photoluminescence spectra analysis results (Figure 7a), the fluorometric lifetime analysis results (Figure 7b), the transient photocurrent intensity variation experimentation analysis results (Figure 7c), the electrochemistry resistance experimentation analysis results (Figure 7d), the UV-Vis absorption spectrum analysis results (Figure 5), the XPS binding energy shift analysis results (Figure 4), radicals trapping experimental analysis results (Figure 8a,b), the EPR spectrogram analysis results (Figure 8c), and the valence band position measurement analysis results (Figure 9) strongly suggested that the degradation mechanism of BLG when using the HZ catalyst was consistent with the charge-transfer mechanism of the direct Z-scheme heterojunction [56,57].

#### 3.2.3. Possible Degradation Pathways of the BLG

**Figure 11:**
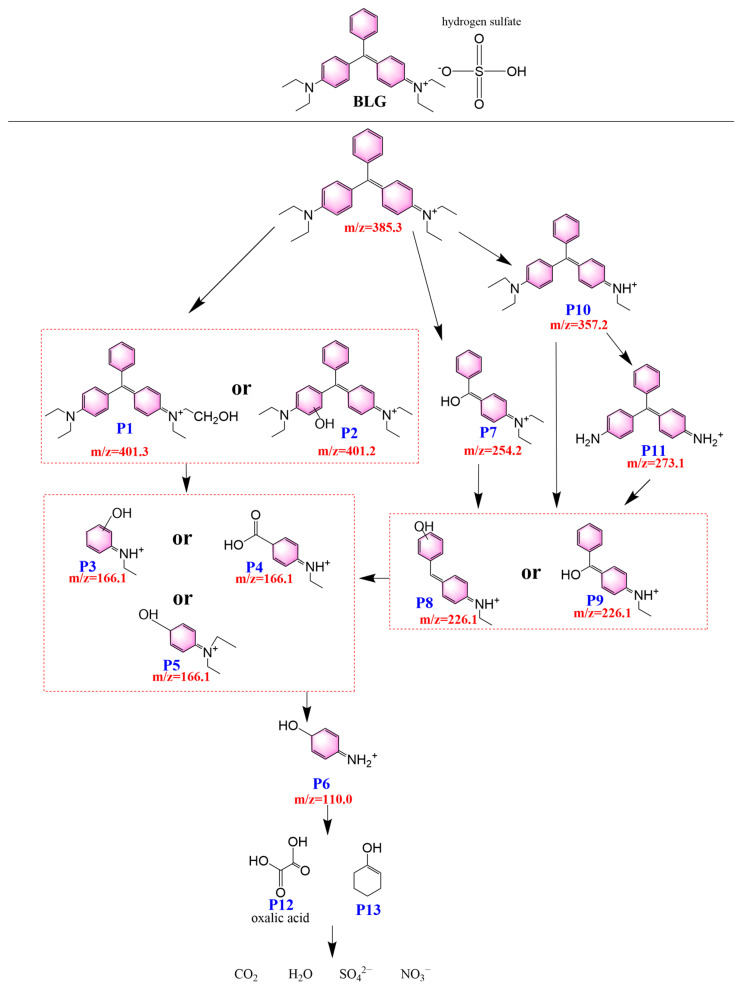
The possible degradation pathways for the BLG under visible light irradiation with the HZ as the catalyst.

According to the literature, this work identified possible degradation routes of the BLG (*m*/*z* = 385.3) and corresponding intermediate products of the BLG. As revealed in Figure 11, three different degradation routes and thirteen intermediate products of the BLG were analyzed.

In the first degradation route of the BLG, the degradation procedure of the BLG could start with aromatic ring hydroxylation; accordingly, the hydroxylated intermediate product P1 (*m*/*z* = 401.3) or P2 (*m*/*z* = 401.2) was formed [58,59]. Subsequently, P1 or P2 underwent dealkylation and oxidative cleavage of the aromatic ring to yield the product P3 (*m*/*z* = 166.1), P4 (*m*/*z* = 166.1) or P5 (*m*/*z* = 166.1) [58,59]. In rapid sequence, the removal of the alkyl group yielded the product P6 (*m*/*z* = 110.0) [59,60]. Low-molecular-weight compound P12 or P13 was formed [61].

In the second degradation route of the BLG, the degradation procedure could operate as the hydroxylation of the BLG, where the intermediate product P7 (*m*/*z* = 254.2) was formed [58,59]. Subsequently, P7 underwent dealkylation to form P8 (*m*/*z* = 226.1) or P9 (*m*/*z* = 226.1) [58,59]. Afterward, the degradation of the aromatic ring through cleavage yielded the product, which had an *m*/*z* value of 166.1.

In the third degradation route of the BLG, P10 (*m*/*z* = 357.2) was firstly generated through the removal of alkyl groups; subsequently, P10 further reacted with oxidic radicals to form P11 (*m*/*z* = 273.1) [58,59,61,62]. P10 or P11 further underwent oxidative cleavage of the aromatic structure to form a product with an *m*/*z* value of 226.1 [58,59]. Eventually, P12 or P13 was further degraded into carbon dioxide (CO_2_), water (H_2_O) and inorganic anions, which included sulfate radical anion (SO_4_^2−^) and nitrate radical anion (NO_3_^−^).

## 4. Conclusions

In conclusion, the direct Z-type HZ catalyst was prepared first using a wet dipping method. ZnBiTmO_4_ remained in the body-centered tetragonal system with a spinel structure, while the space group of ZnBiTmO_4_ was I41/AMD, and the crystal cell parameters of ZnBiTmO_4_ were a = b = 14.798828 Å, c = 9.632210 Å. Ho_2_BiNbO_7_ remained in the cubic system and the pyrochlore structure, while the space group of Ho_2_BiNbO_7_ was Fd3m, and the crystal cell parameters of Ho_2_BiNbO_7_ were a = b = c = 10.622091 Å. The bandgap width of Ho_2_BiNbO_7_, ZnBiTmO_4_ or HZ was computed to be 1.869 eV, 1.777 eV or 1.732 eV. The experimental results revealed that there was an excellent Z-type electric charge migration mechanism between ZnBiTmO_4_ and Ho_2_BiNbO_7_. Accordingly, the HZ catalyst not only immensely enhanced the separation efficiency of the photogenerated electrons and photogenerated holes but also kept strong oxidic capability and strong reductive capability. The experimental results displayed that the HZ photocatalyst had an excellent photocatalytic activity during the degradation process of the BLG. In particular, the degradation rate of the BLG when using the HZ photocatalyst was found to be 99.47%, and the removal rate of the TOC-C was found to be 98.26% when using the HZ photocatalyst under VILIIR. The HZ photocatalyst possessed higher photocatalytic activity than the Ho_2_BiNbO_7_, ZnBiTmO_4_, or N-T photocatalysts. The degradation rate of the BLG when using the HZ photocatalyst was 1.27 times, 1.15 times and 2.91 times higher than that when using the Ho_2_BiNbO_7_ photocatalyst, ZnBiTmO_4_ photocatalyst, or N-T photocatalyst under VILIIR, respectively. The mineralization removal rate of TOC-C in the catalytical degradation of BLG when employing the HZ photocatalyst was 1.31 times, 1.19 times and 3.14 times higher than that when employing the Ho_2_BiNbO_7_ photocatalyst, ZnBiTmO_4_ photocatalyst, or N-T photocatalyst under VILIIR, respectively. Radicals-catching experiments and EPR measurement experiments confirmed that the HZ photocatalyst could generate multiple reactive species, such as •O_2_^−^, •OH and h^+^, in the process of catalytical degradation of the BLG. The descending order of the oxidizing capacity for the three radicals was as follows: •OH > •O_2_^−^ > h^+^. The descending order of the photocatalytic activity for the four photocatalysts was as follows: HZ photocatalyst > ZnBiTmO_4_ > Ho_2_BiNbO_7_ > N-T. The intermediate degradation products of the BLG were detected when using the HZ photocatalyst in the process of catalytical degradation for the BLG, while the reliability, reusability, and stability of the HZ photocatalyst were proven by quintic cyclic degradation experiments of the BLG. This work developed the degradation pathways and the degradation mechanism of the BLG when using the HZ photocatalyst under VILIIR. Ultimately, this study also systematically determined the catalytical degradation mechanism and degradation route of the BLG. These discoveries may allow the HZ catalyst to be widely applied for the catalytic degradation of the organic dye contaminants from dye printing wastewater.

## Data Availability

The original contributions presented in this study are included in the article/Supplementary Material. Further inquiries can be directed to the corresponding authors.

## Supplementary Materials

The following supporting information can be downloaded at https://www.mdpi.com/article/10.3390/nano16150951/s1, Figure S1: The research process flowchart for each stage of this study; Figure S2: (a) The XRD pattern and the Pawley refinement result, and (b) the atomic architecture of Ho_2_BiNbO7; Figure S3: (a) The XRD pattern and the Pawley refinement result, and (b) the atomic architecture of ZnBiTmO_4_; Figure S4: HRTEM image of HZ; Figure S5: The N_2_ sorption isotherms of of (a) HZ, (b) Ho_2_BiNbO_7_, and (c) ZnBiTmO_4_; Figure S6: The XPS patterns of the unused HZ and the used HZ; Figure S7: (a) The degradation curves of the BLG, (b) the kinetic curves for the BLG concentration variation, and (c) the removal efficiencies and kinetic constants of the BLG concentration after five cyclical tests for degrading the BLG; (d) The mineralization curves for removing TOC concentration, (e) the kinetic curves for TOC concentration variation, and (f) the mineralization efficiencies and kinetic constants for TOC concentration after five cyclical degradation tests for removing TOC concentration with HZ as catalyst under visible light irradiation; Figure S8: (a) The degradation curves of the phenol, (b) The mineralization curves for removing TOC concentration with Ho_2_BiNbO_7_, ZnBiTmO_4_, HZ or N-T as photocatalyst under visible light irradiation; Table S1: The concentration variation of the brilliant green by the adsorption effect of the HZ under dark conditions; Table S2: Structural properties of Ho_2_BiNbO_7_ fabricated with the rapid microwave-assisted high temperature treatment method; Table S3: Structural properties of ZnBiTmO_4_ fabricated with the homogeneous precipitation assisted heat treatment method; Table S4: Comparison of photocatalytic performance of the HZ photocatalyst with other reported photocatalysts.

## References

[1] Kaur S., Sharma S., Umar A., Singh S., Mehta S., Kansal S. (2017). Solar light driven enhanced photocatalytic degradation of brilliant green dye based on ZnS quantum dots. Superlattices Microstruct..

[2] Ameta K., Sharma J., Solanki V. (2022). Photocatalytic mineralization of brilliant green dye using bimetallic CeCuO_3_ nanoparticles in LEDs irradiations: A green and economically viable approach. Results Chem..

[3] Donga C., Ratshiedana R., Kuvarega A., Masunga N., Vallabhapurapu V., Mbule P. (2025). Photocatalytic degradation of organic pollutants in wastewater using magnetic functionalized reduced graphene oxide nanocomposites. A review. Talanta.

[4] Nguyen T., Le T., Hoang C., Nguyen H., Huynh T. (2024). Silver-modification of ZnO thin films deposited on Zn-electroplated Cu substrate by thermal shock method for the enhanced photocatalytic performance. J. Taibah Univ. Sci..

[5] Meng K., Zhang J., Cheng B., Ren X., Xia Z., Xu F., Zhang L., Yu J. (2024). Plasmonic near-infrared-response S-scheme ZnO/CuInS_2_ photocatalyst for H_2_O_2_ production coupled with glycerin oxidation. Adv. Mater..

[6] Ratshiedana R., Mafa P., Kuvarega A. (2025). Piezo-photocatalytic applications of BiOBr-based materials for organic pollutants degradation. J. Environ. Chem. Eng..

[7] Xu J., Xi R., Xu X., Zhang Y., Feng X., Fang X., Wang X. (2020). A_2_B_2_O_7_ pyrochlore compounds: A category of potential materials for clean energy and environment protection catalysis. J. Rare Earths.

[8] Lv D., Zhang D., Sun Q., Wu J., Zhang L., Pu X., Ma H., Dou J. (2016). A novel method for the synthesis of BiOCl/Bi_2_Sn_2_O_7_ heterojunction photocatalysts with enhanced visible light photocatalytic properties. Nanotechnology.

[9] Lang J., Li C., Wang S., Lv J., Su Y., Wang X., Li G. (2015). Coupled heterojunction Sn_2_Ta_2_O_7_@SnO_2_: Cooperative promotion of effective electron hole separation and superior visible-light absorption. ACS Appl. Mater. Interfaces.

[10] Derkaoui K., Bencherifa I., Elfiad A., Mebdoua Y., Belkhettab I., Boukhouidem K., Benredouane S., Hadjersi T., Manseri A., Kechouane M. (2025). Unveiling the optical and dielectric properties of MnFe_2_O_4_: A high-performance visible-light photocatalyst for sustainable rhodamine B degradation. J. Electron. Mater..

[11] Muthukrishnaraj A., Vadivel S., Joni I., Balasubramanian N. (2015). Development of reduced graphene oxide/CuBi_2_O_4_ hybrid for enhanced photocatalytic behavior under visible light irradiation. Ceram. Int..

[12] Phuruangrat A., Yayapao O., Thongtem T., Thongtem S. (2014). Preparation, characterization and photocatalytic properties of Ho doped ZnO nanostructures synthesized by sonochemical method. Superlattices Microstruct..

[13] Zhu X., Liu Z., Fang J., Wu S., Xu W. (2013). Synthesis and characterization of mesoporous Bi/TiO_2_ nanoparticles with high photocatalytic activity under visible light. J. Mater. Res..

[14] Yang J., Zhang X., Wang C., Sun P., Wang L., Xia B., Liu Y. (2012). Solar photocatalytic activities of porous Nb-doped TiO_2_ microspheres prepared by ultrasonic spray pyrolysis. Solid State Sci..

[15] Varadharajan K., Singaram B., Mani R., Jeyaram J. (2016). Enhanced visible light photocatalytic activity of Ag and Zn doped and codoped TiO_2_ nanoparticles. J. Clust. Sci..

[16] Karnchana N., Phuruangrat A., Thongtem S., Thongtem T. (2021). Combustion synthesis and characterization of visible-light-driven Tm-doped ZnO nanoparticles used for photodegradation of methylene blue. Dig. J. Nanomater. Biostructures.

[17] Luangwanta T., Thiraphatchotiphum C., Kaowphong S. (2025). Microwave-synthesized BiOCl-Bi_4_O_5_Br_2_ solid-solution photocatalysts with tunable band structures for efficient visible-light-driven removal of Cr(VI) As(III), and bisphenol A. J. Water Process Eng..

[18] Fattah W., Gobara M., El-Hotaby W., Mostafa S., Ali G. (2016). Coating stainless steel plates with Ag/TiO_2_ for chlorpyrifos decontamination. Mater. Res. Express.

[19] Jo W., Natarajan T. (2015). Influence of TiO_2_ morphology on the photocatalytic efficiency of direct Z-scheme g-C_3_N_4_/TiO_2_ photocatalysts for isoniazid degradation. Chem. Eng. J..

[20] Phu N., Vu P., Dung D., Bich D., Oanh L., Hoang L., Hung N., Hai P. (2019). Temperature-dependent preparation of bismuth pyrostannate Bi_2_Sn_2_O_7_ and its photocatalytic characterization. Mater. Chem. Phys..

[21] Ai J., Lin C., Liao W., Hu C., Zhou T., Luo S., Cheng L., Chen Z., Yang Y., Zhang Y. (2019). Shape-controlled synthesis of bare ZnO nanomaterials with improved photoactivity and photostability. Mater. Sci. Technol..

[22] Jo W., Lee J., Natarajan T. (2016). Fabrication of hierarchically structured novel redox-mediator-free ZnIn_2_S_4_ marigold flower/Bi_2_WO_6_ flower-like direct Z-scheme nanocomposite photocatalysts with superior visible light photocatalytic efficiency. Phys. Chem. Chem. Phys..

[23] Zhang Z., Goodall J., Morgan D., Brown S., Clark R., Knowles J., Mordan N., Evans J., Carley A., Bowker M. (2009). Photocatalytic activities of N-doped nano-titanias and titanium nitride. J. Eur. Ceram. Soc..

[24] Wang J., Zou Z., Ye J., Pan W., Gong J., Zhang L., Chen L. (2003). Synthesis, structure and photocatalytic property of a new hydrogen evolving photocatalyst Bi_2_InTaO_7_. Functionally Graded Materials VII.

[25] Kudo A., Kato H., Nakagawa S. (2000). Water splitting into H_2_ and O_2_ on new Sr_2_M_2_O_7_ (M = Nb and Ta) photocatalysts with layered perovskite structures: Factors affecting the photocatalytic activity. J. Phys. Chem. B.

[26] Du H., Luan J. (2012). Synthesis, characterization and photocatalytic activity of new photocatalyst CdBiYO_4_. Solid State Sci..

[27] Hao L., Luan J. (2024). Visible light-driven direct Z-Scheme Ho_2_SmSbO_7_/YbDyBiNbO_7_ heterojunction photocatalyst for efficient degradation of fenitrothion. Molecules.

[28] Mortazavi-Derazkola S., Salavati-Niasari M., Amiri O., Abbasi A. (2017). Fabrication and characterization of Fe_3_O_4_@SiO_2_@TiO_2_@Ho nanostructures as a novel and highly efficient photocatalyst for degradation of organic pollution. J. Energy Chem..

[29] Singh J., Roychoudhury A., Srivastava M., Solanki P., Lee D., Lee S., Malhotra B. (2014). A dual enzyme functionalized nanostructured thulium oxide based interface for biomedical application. Nanoscale.

[30] Luan J., Li Z., Yao Y., Wang J., Hao L. (2025). Metronidazole degradation via visible light-driven Z-Scheme BiTmDySbO_7_/BiEuO_3_ heterojunction photocatalyst. Sustainability.

[31] Sulym I., Wisniewska M., Storozhuk L., Terpilowski K., Sternik D., Borysenko M., Derylo-Marczewska A. (2022). Investigation of surface structure, electrokinetic and stability properties of highly dispersed Ho_2_O_3_-Yb_2_O_3_/SiO_2_ nanocomposites. Appl. Nanosci..

[32] Hou R., Pang K., Bai Z., Feng Z., Ye D., Guo Z., Kong L., Bai J., Li W. (2021). Study on carboxyl groups in direct liquefaction of lignite: Conjoint analysis of theoretical calculations and experimental methods. Fuel.

[33] Mohamed H., Khalil A., Hkiri K., Ayaz M., Usman A., Sadiq A., Ullah F., Hussain I., Maaza M. (2023). Phyto-fabrication of ultrafine nanoscale holmium oxide HT-Ho_2_O_3_ NPs and their biomedical potential. RSC Adv..

[34] Cadis A., Rus F., Goncalves J., Ivanovici M. (2023). Preparing a Ca-Bi-O system by the precipitation method and studying its intermediate structural properties for applications in water treatment. Inorganics.

[35] Ribeiro J., Correia F., Salvador P., Rebouta L., Alves L., Alves E., Barradas N., Mendes A., Tavares C. (2019). Compositional analysis by RBS, XPS and EDX of ZnO: Al, Bi and ZnO: Ga, Bi thin films deposited by d.c. magnetron sputtering. Vacuum.

[36] Szatmari A., Bortnic R., Dragoiu T., Hategan R., Barbu-Tudoran L., Tiusan C., Lucacel-Ciceo R., Dudric R., Tetean R. (2025). XPS on Co_0.95_R_0.05_Fe_2_O_4_ nanoparticles with R = Gd or Ho. Appl. Sci..

[37] Lin L., Ali K. (2024). Photocatalytic degradation of organic compounds in dye wastewater by Fe^3+^ doped nano-ZnO/TiO_2_ composite photocatalyst. Indian J. Chem. Technol..

[38] Chen F., Li Y., Zhou M., Gong X., Gao Y., Cheng G., Ren S., Han D. (2023). Smart multifunctional direct Z-scheme In_2_S_3_@PCN-224 heterojunction for simultaneous detection and photodegradation towards antibiotic pollutants. Appl. Catal. B Environ..

[39] Nowak M., Kauch B., Szperlich P. (2009). Determination of energy band gap of nanocrystalline SbSI using diffuse reflectance spectroscopy. Rev. Sci. Instrum..

[40] Nagatani H., Suzuki I., Kita M., Tanaka M., Katsuya Y., Sakata O., Omata T. (2015). Structure of β-AgGaO_2_; ternary I-III-VI2 oxide semiconductor with a wurtzite-derived structure. J. Solid State Chem..

[41] Sakthivel S., Shankar M., Palanichamy M., Arabindoo B., Bahnemann D., Murugesan V. (2004). Enhancement of photocatalytic activity by metal deposition: Characterisation and photonic efficiency of Pt, Au and Pd deposited on TiO_2_ catalyst. Water Res..

[42] Marugán J., Hufschmidt D., Sagawe G., Selzer V., Bahnemann D. (2006). Optical density and photonic efficiency of silica-supported TiO_2_ photocatalysts. Water Res..

[43] Luan J., Ma B., Yao Y., Liu W., Niu B., Yang G., Wei Z. (2022). Synthesis, Performance Measurement of Bi_2_SmSbO_7_/ZnBiYO_4_ Heterojunction Photocatalyst and Photocatalytic Degradation of Direct Orange within Dye Wastewater under Visible Light Irradiation. Materials.

[44] Devi V., Ravi G., Velchuri R., Muniratnam N., Prasad G., Vithal M. (2013). Preparation, Characterization, Photocatalytic Activity and Conductivity Studies of YLnTi_2_O_7_ (Ln = Nd, Sm, Eu and Gd). Trans. Indian Ceram. Soc..

[45] Balakrishnan G., Velavan R., Batoo K., Raslan E. (2020). Microstructure, optical and photocatalytic properties of MgO nanoparticles. Results Phys..

[46] Buschmann V., Ermilov E., Koberling F., Loidolt-Krüeger M., Breitlow J., Kooiman H., Los J., van Willigen J., Caldarola M., Fognini A. (2023). Integration of a superconducting nanowire single-photon detector into a confocal microscope for time-resolved photoluminescence (TRPL)-mapping: Sensitivity and time resolution. Rev. Sci. Instrum..

[47] Chen J., Kim S., Ren X., Jung H., Park N. (2019). Effect of bidentate and tridentate additives on the photovoltaic performance and stability of perovskite solar cells. J. Mater. Chem. A.

[48] Shen Y., Liu Y., Xi X., Nie Z. (2024). Anchoring Cu_2_O onto WO_3_ by adsorption-ascorbic acid reduction: A p-n junction with improved photocatalytic and photoelectrochemical properties. Opt. Mater..

[49] Ibeniaich M., Elansary M., Minaoui K., Mouhib Y., Haj Y., Belaiche Y., Oulhakem O., Iffer E., Ferdi C., Lemine O. (2024). Exploring the effect of Hf (IV) doping in spinel ferrite CoHfxFe_2-x_O_4_ on magnetic properties, electrochemical impedance, and photocatalytic activity: In-depth structural study. J. Mol. Struct..

[50] Wang Y., Li X., Liu S., Liu Y., Kong T., Zhang H., Duan X., Chen C., Wang S. (2022). Roles of catalyst structure and gas surface reaction in the generation of hydroxyl radicals for photocatalytic oxidation. ACS Catal..

[51] Zhuang Y., Luan J. (2020). Improved photocatalytic property of peony-like InOOH for degrading norfloxacin. Chem. Eng. J..

[52] Xu S., Gong S., Jiang H., Shi P., Fan J., Xu Q., Min Y. (2020). Z-scheme heterojunction through interface engineering for broad spectrum photocatalytic water splitting. Appl. Catal. B Environ..

[53] Yuan Y., Guo R., Hong L., Ji X., Lin Z., Li Z., Pan W. (2021). A review of metal oxide-based Z-scheme heterojunction photocatalysts: Actualities and developments. Mater. Today Energy.

[54] Shabbir A., Sardar S., Mumtaz A. (2024). Mechanistic investigations of emerging type-II, Z-scheme and S-scheme heterojunctions for photocatalytic applications—A review. J. Alloys Compd..

[55] Nkudede E., Xing Q., Pang Z., Liu N., Yan X., Sulemana H., Yusuf B., Di J., Yin S., Xia J. (2024). A novel Type-II Bi-based oxyhalide: Bi_4_O_5_I_2_/BiOBr microspheres for improved photocatalytic performance. Mater. Sci. Semicond. Process..

[56] Zhang J., Hu Y., Jiang X., Chen S., Meng S., Fu X. (2014). Design of a direct Z-scheme photocatalyst: Preparation and characterization of Bi_2_O_3_/g-C_3_N_4_ with high visible light activity. J. Hazard. Mater..

[57] Miao J., Yang Y., Cui P., Ru C., Zhang K. (2024). Improving charge transfer beyond conventional heterojunction photoelectrodes: Fundamentals, strategies and applications. Adv. Funct. Mater..

[58] Liu L., Wang L., Zhou L., Zhang Y., Yang X., Han Z., Yu Z. (2025). Highly efficient visible-light degradation of brilliant green over Z-scheme BiOCl/Sn-BDC heterojunction photocatalysts: Mechanism and phytotoxicity assessment. J. Alloys Compd..

[59] Liu J., Guo C., Lu Y., Liu H., Jia M., Zhang J., Jin R., Qiao Y., Ma R., Wu Z. (2023). Experimental and theoretical study on the photocatalytic degradation of brilliant green dye by N-doped Zn_2_GeO_4_. Chem. Eng. Sci..

[60] Khan Z., Shah N., Iqbal J., Khan A., Imran M., Alshehri S., Muhammad N., Sayed M., Ahmad N., Kousar A. (2020). Biomedical and photocatalytic applications of biosynthesized silver nanoparticles: Ecotoxicology study of brilliant green dye and its mechanistic degradation pathways. J. Mol. Liq..

[61] Qi C., Chen H., Xu C., Xu Z., Chen H., Yang S., Li S., He H., Sun C. (2020). Synthesis and application of magnetic materials-barium ferrite nanomaterial as an effective microwave catalyst for degradation of brilliant green. Chemosphere.

[62] Han Z., Gu X., Wang S., Liu L., Wang Y., Zhao Z., Yu Z. (2020). Time-resolved in situ monitoring of photocatalytic reactions by probe electrospray ionization mass spectrometry. Analyst.

