# Supplementary material for: Synthesis and Property Detection of the Ho2BiNbO7/ZnBiTmO4 Composite Catalyst for Photocatalytic Degradation of Brilliant Green
Source: Nanomaterials (Basel). 2026 Aug 2;16(15):951. doi: 10.3390/nano16150951 (PMC13468221; doi:10.3390/nano16150951)
Supplement: Supplementary file 1 [file nanomaterials-16-00951-s001.zip › nanomaterials-4456194-supplementary.pdf]

Synthesis and Property Detection of the $Ho_2BiNbO_7/ZnBiTmO_4$ Composite Catalyst for Photocatalytic Degradation of Brilliant Green

Jingfei Luan [1,2,*,†] and Boyang Liu [1,†]

1 School of Physics, Changchun Normal University, Changchun 130032, China; boyangliu152@outlook.com
2 State Key Laboratory of Pollution Control and Resource Reuse, School of the Environment, Nanjing University, Nanjing 210093, China
* Correspondence: jfluan@nju.edu.cn; Tel.: +86-199-5193-9498
† These authors contributed equally to this work.

Table S1. The concentration variation of the brilliant green by the adsorption effect of the HZ under dark conditions.

Dark adsorption time (min)	-63	-54	-45	-36	-27	-18	-9	0
Brilliant green concentration (mM)	0.03200	0.03152	0.03098	0.03041	0.02994	0.02957	0.02954	0.02952

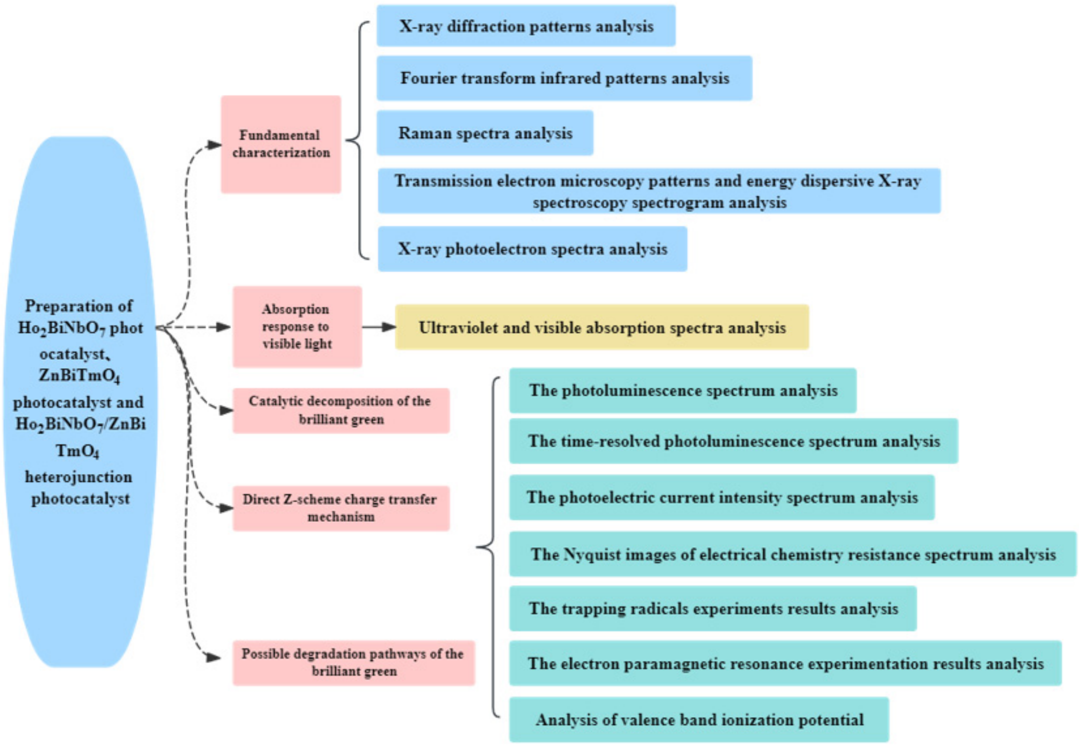

Figure S1. The research process flowchart for each stage of this study.

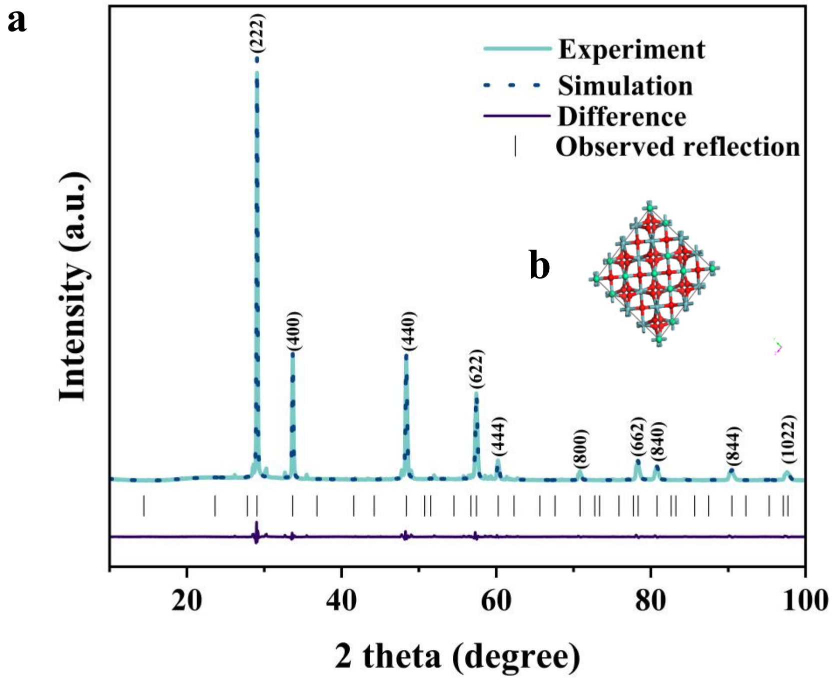

Figure S2. **(a)** The XRD pattern and the Pawley refinement result, and **(b)** the atomic architecture of Ho_2BiNbO_7.

Table S2. Structural properties of Ho_2BiNbO_7 fabricated with the rapid microwave-assisted high temperature treatment method.

**Atom**	x	y	z	**Occupation Factor**
Ho	0	0	0	1
Bi	0.5	0.5	0.5	0.5
Nb	0.5	0.5	0.5	0.5
O(1)	-0.125	0.125	0.125	1
O(2)	0.125	0.125	0.125	1

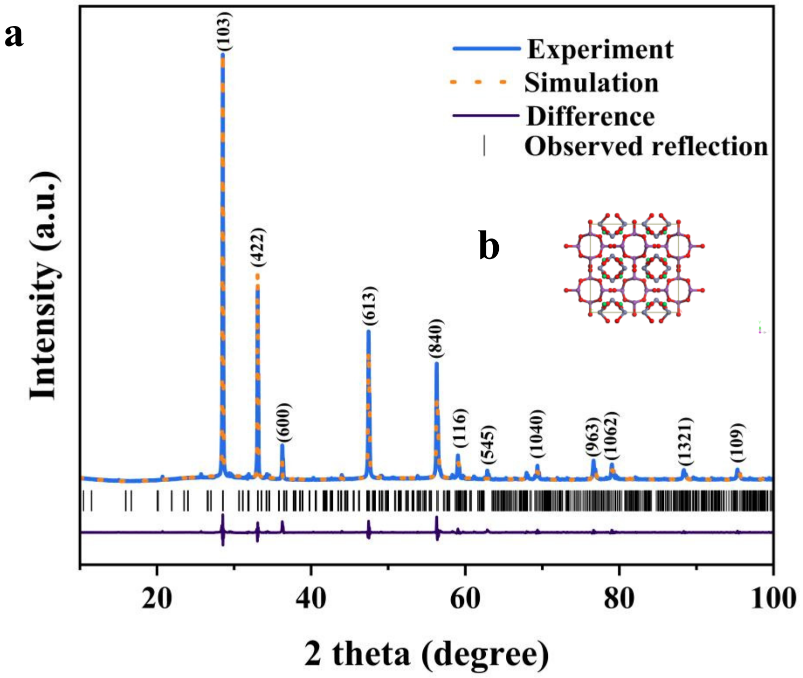

Figure S3. (a) The XRD pattern and the Pawley refinement result, and **(b)** the atomic architecture of $ZnBiTmO_4$.

Table S3. Structural properties of $ZnBiTmO_4$ fabricated with the homogeneous precipitation assisted heat treatment method.

**Atom**	x	y	z	**Occupation Factor**
Zn	0.39003	0.00000	0.00000	1
Bi	0.00000	0.11132	0.22252	1
Tm	0.15752	0.09252	0.87500	1
O(1)	0.00000	0.97728	0.30179	1
O(2)	0.15267	0.05286	0.12528	1
O(3)	0.39217	0.14216	0.12500	1

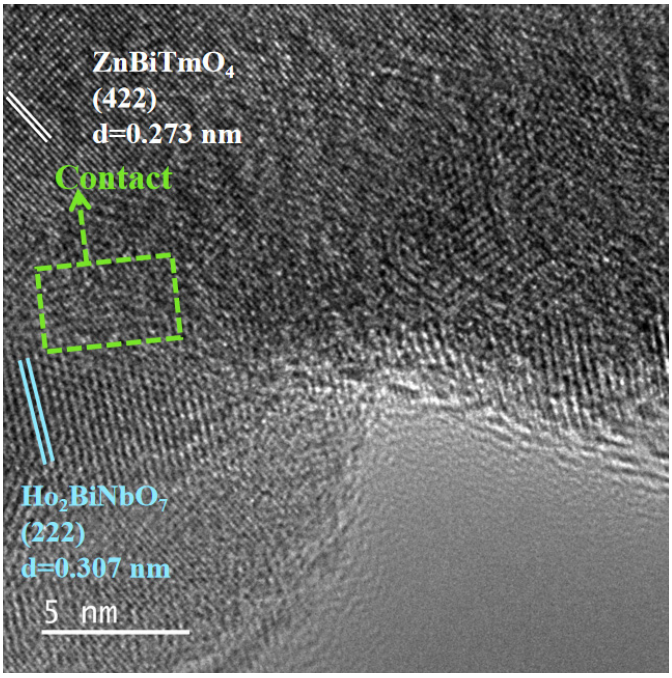

Figure S4. HRTEM image of HZ.

a

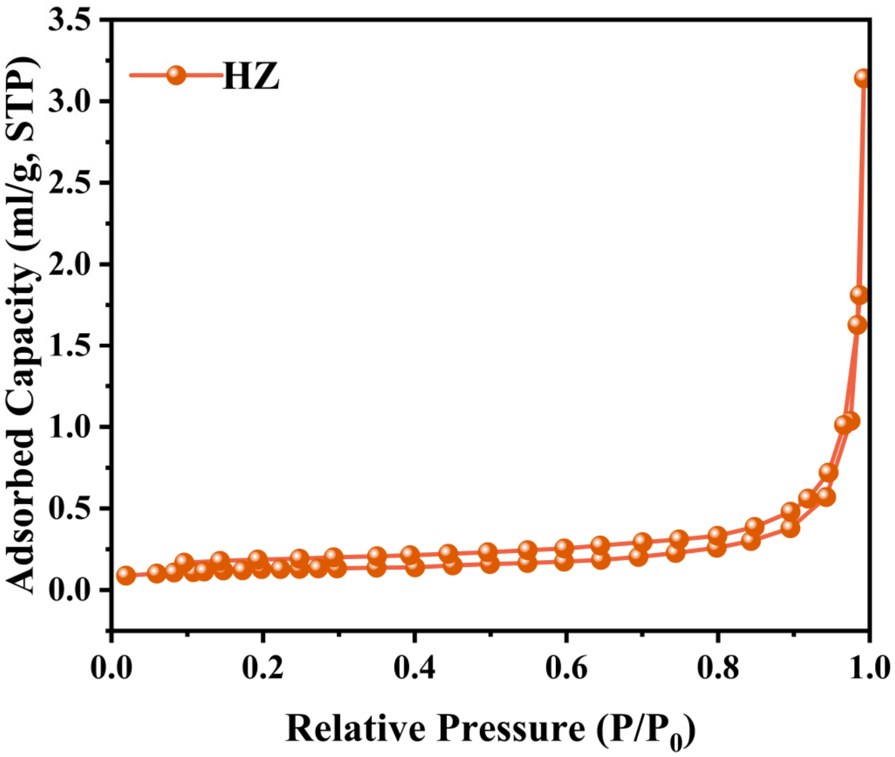

b

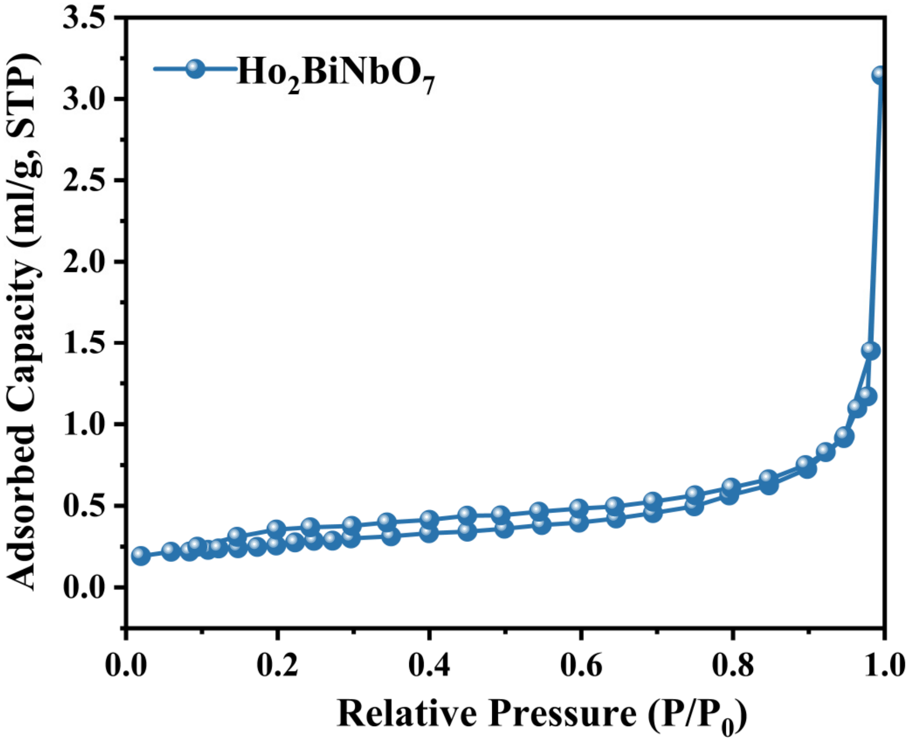

c

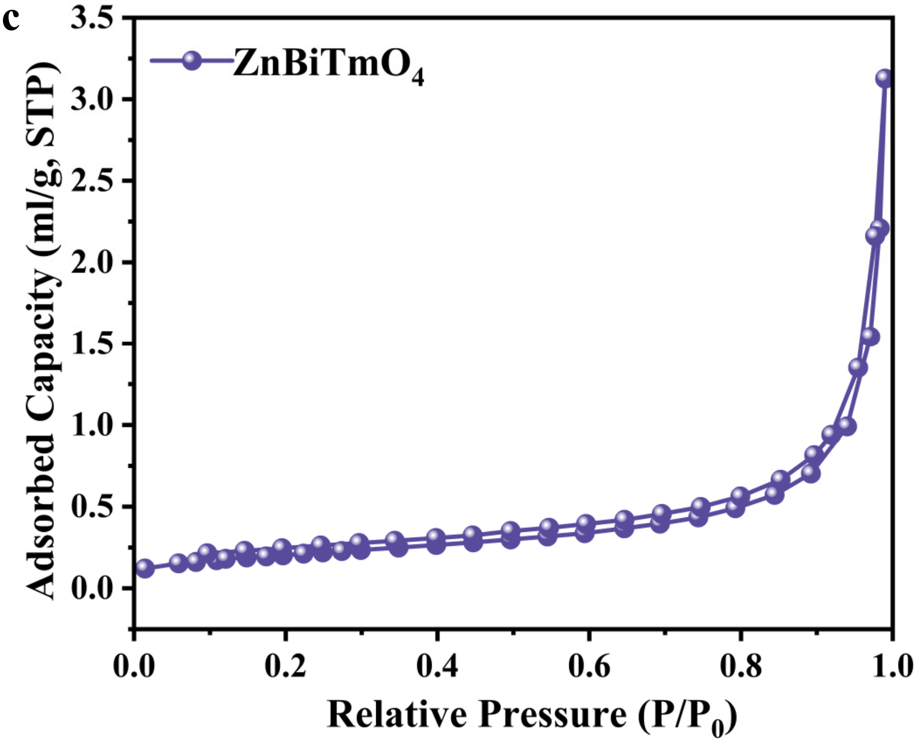

Figure S5. The N_2 sorption isotherms of of **(a)** HZ, **(b)** Ho_2BiNbO_7, and **(c)** $ZnBiTmO_4$.

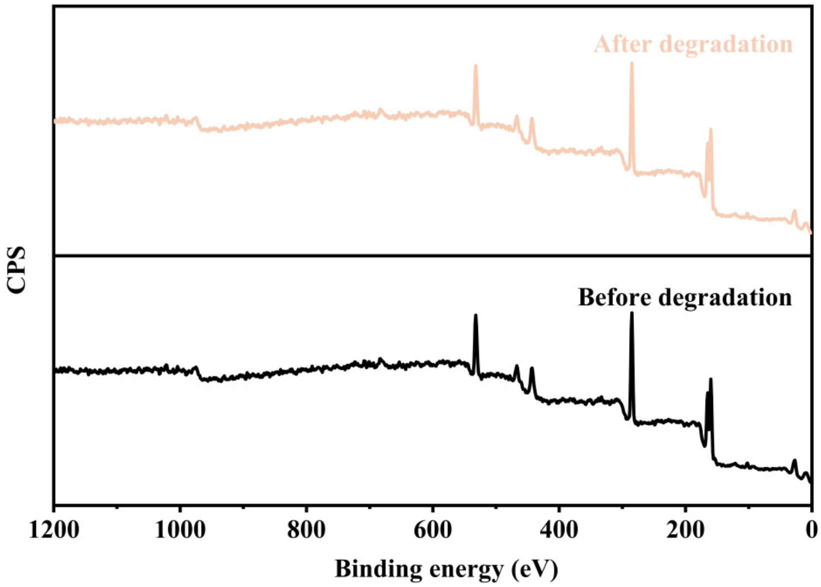

Figure S6. The XPS patterns of the unused HZ and the used HZ.

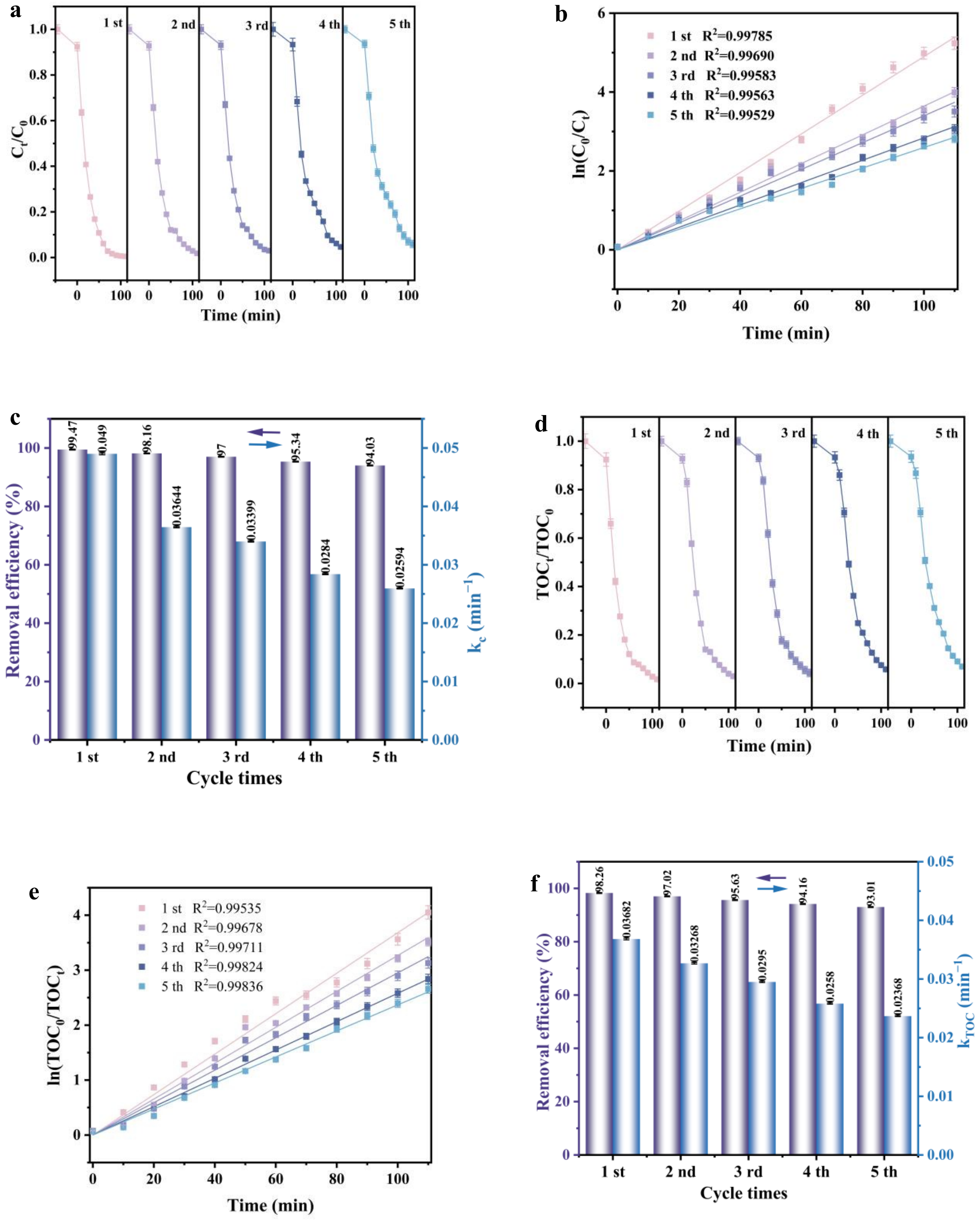

Figure S7. (a) The degradation curves of the BLG, **(b)** the kinetic curves for the BLG concentration variation, and **(c)** the removal efficiencies and kinetic constants of the BLG concentration after five cyclical tests for degrading the BLG; **(d)** The mineralization curves for removing TOC concentration, **(e)** the kinetic curves for TOC concentration variation, and **(f)** the mineralization efficiencies and kinetic constants for TOC concentration after five cyclical degradation tests for removing TOC concentration with HZ as catalyst under visible light irradiation.

Table S4. Comparison of photocatalytic performance of the HZ photocatalyst with other reported photocatalysts.

**Photocatalyst**	**Pollutant**	**Irradiation Time (min)**	**Removal Rate**	**Reference**
$Bi_2SmSbO_7/ZnBiYO_4$ heterojunction photocatalyst	direct orange	160	99.10%	[39]
$MnFe_2O_4$	Rhodamine B	90	68.51%	[10]
$YGdTi_2O_7$	methylene blue	180	66.00%	[40]
$Ho_2BiNbO_7/ZnBiTmO_4$ heterojunction photocatalyst	brilliant green	110	99.47%	This study

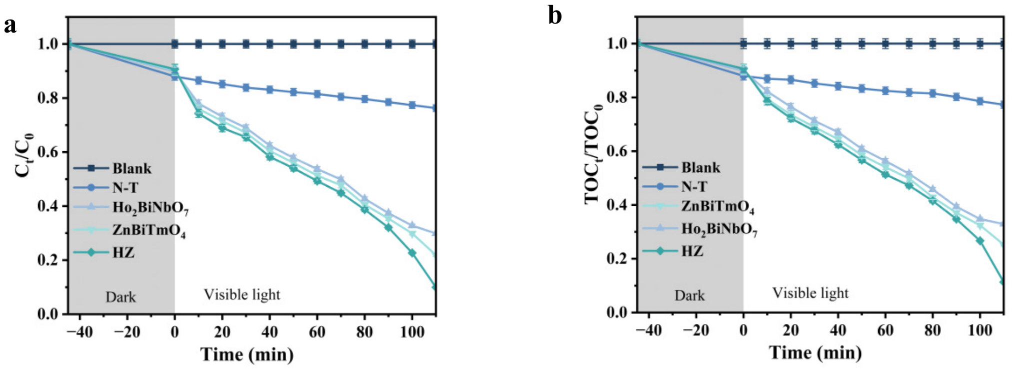

Figure S8. (a) The degradation curves of the phenol, **(b)** The mineralization curves for removing TOC concentration with Ho_2BiNbO_7, $ZnBiTmO_4$, HZ or N-T as photocatalyst under visible light irradiation.
